# Nature is nurture: a scoping review of nature exposure as an equigenic intervention on children’s psychological health

**DOI:** 10.3389/fpsyg.2026.1731222

**Published:** 2026-04-10

**Authors:** Keira I. Denker, Andrea Faber Taylor

**Affiliations:** 1 Department of Psychology, University of Illinois Urbana-Champaign, Urbana, IL, United States; 2 Department of Crop Sciences, University of Illinois Urbana-Champaign, Urbana, IL, United States

**Keywords:** child development, equigenesis, greenspace, literature review, mental health, nature exposure, psychological development, socioeconomic status

## Abstract

Research indicates that exposure to nature has positive effects on the mental health and psychological development of children. Children from less advantaged groups are in particular need of support in these domains, often experiencing poorer mental health and delays in psychological development. Thus, recent research has begun exploring the potential for nature experiences to have an “equigenic effect” on children’s psychological health, boosting disadvantaged groups to achieve outcomes similar to advantaged groups. This scoping review presents a light review of the literature examining equigenic effects of nature exposure on health in adults and children and a deeper analysis of studies focused on children’s mental health and psychological development. A search was conducted in EBSCO, PubMed, and Scopus, and a total of 123 empirical articles were included in the review after screening. The results reveal a growing body of evidence of an equigenic effect of nature exposure for adults and children; fewer studies, however, have focused on children. Among 24 studies comparing disadvantaged to advantaged children (ages 0–18), 19 of them demonstrated at least one positive finding in support of equigenesis, but there were also mixed findings. Themes in the literature regarding nature exposure and possible mechanisms underlying the potential equigenic relationship between nature exposure and children’s psychological health are explored. Future research suggestions and implications for increasing nature exposure in children’s lives through daily routines, nature-based learning, and improving equitable access are also discussed. This review presents evidence that exposure to nature may be an effective intervention to specifically support children living with disadvantage, promoting greater equity in psychological well-being.

## 1 Introduction

Exposure to nature has numerous positive effects on both the physical and psychological health of adults and children (for review see Jimenez et al., 2021). Specifically for children, some of the known benefits for mental health and psychological development include reduced behavioral difficulties and risk of psychological disorders as well as improved attention, emotional regulation, self-esteem, social behavior, life satisfaction, and reading and math skills (e.g., see reviews Langelier et al., 2025; Lomax et al., 2024; Moll et al., 2022; Tillmann et al., 2018). Positive psychological health during childhood is crucial, as childhood is a key period for development and provides a foundation for well-being in adulthood (Bethell et al., 2019).

However, it has been demonstrated that children living in disadvantaged circumstances—measured by income, parental education, minority status, etc.—experience worse mental health or delays in psychological development, likely due to the greater number of daily stressors they experience (Businelle et al., 2014; Yoshikawa et al., 2012). Altering a child’s physical environment may mitigate these disparities. *Equigenesis*, coined by Dr. Richard Mitchell, is the concept that the characteristics of physical environments may impact human health and functioning differentially, acting as a *leveling force*, benefiting disadvantaged populations more and promoting equity in both the physical and psychological domains (Mitchell, 2013). This has been referred to as the *equigenesis hypothesis* by studies examining the leveling effects of *equigenic environments*.

Given the well-established link between exposure to nature and positive health outcomes, greenspaces may act as equigenic environments, potentially boosting disadvantaged children’s psychological health to levels similar to their advantaged peers. However, the concept of equigenesis is relatively new, and there is quite a bit more to learn regarding if, how, and for whom equigenesis occurs. Few studies have synthesized the existing evidence for the equigenesis hypothesis, especially with regards to children’s mental health and psychological development. One review, focused on reducing inequities in children’s mental health in relation to characteristics of physical environments, has proposed that greenspace may be an important characteristic (Alderton et al., 2019). The current review extends that work, employing a scoping approach, mapping the breadth and depth of research on this topic to (1) fully explore the state of the literature on the potential equigenic effects of nature exposure on general health in adults and children and (2) highlight benefits for children’s psychological health, identifying types of evidence, potential mechanisms, and gaps in the literature (Arksey and O’Malley, 2005; Colquhoun et al., 2014). Our research questions are as follows:

1 What is the current state of the literature comparing groups of differing advantage when testing for the effects of nature exposure on health outcomes (physical and psychological) across all ages?
2 What are the findings among studies comparing groups of differing advantage when testing for the effects of nature exposure on the mental health and psychological development outcomes for children?
3 What are the mechanisms underlying the potential equigenic relationship between nature exposure and mental health and psychological development outcomes for children?
4 What are the implications of such findings and what should future research explore?

From this point forward, we will refer to the independent variable as either *nature exposure* or *greenspace* as appropriate based on studies’ use of these constructs. We have presented the many facets of this construct in Supplementary Table 1.

## 2 Methods

### 2.1 Scope

#### 2.1.1 Initially casting a broad net – All things nature, health, and equigenesis

Testing for equigenesis—comparing groups of differing advantage when testing for effects of nature exposure on health—is a relatively new field of study. While there are proportionately few studies explicitly testing this concept, some of the most compelling and well-known evidence of nature exposure’s equigenic effects has come from studies comparing nature exposure and physical health outcomes for adults of differing advantage levels (for review see Rigolon et al., 2021). Thus, in this review, though our goal is to understand the current evidence and future research needed to support children’s psychological health, we have started with a broader net, gathering studies of nature exposure’s equigenic effect and including all ages as well as both physical and psychological health. Beginning with a broad overview of the adult literature contextualizes the findings of equigenic effects with children and provides additional insights into theoretical underpinnings and potential mechanisms (e.g., Mitchell, 2013; Mitchell et al., 2015). This is particularly important given that, to our knowledge, few existing reviews synthesize equigenic evidence—especially recent findings—related to mental health. Physical health outcomes are also relevant in that they can impact psychological health outcomes (Canadian Mental Health Association, 2008).

#### 2.1.2 Narrowing the pool – Only children and psychological health

From the initial set of included articles, we selected a subset focused on the variables of interest: *children* (ages 0–18 years) and constructs of *mental health* (depression, life satisfaction, etc.) or *psychological development* (attention, memory, socioemotional functioning, etc.). See Supplementary Table 1 for operational definitions of all constructs. We evaluated those studies’ findings for evidence of an equigenic effect. Furthermore, we looked for indications of potential mechanisms underlying any equigenic effects detected in the studies. We also noted gaps in the literature—research that still needs to be conducted to test the idea that nature exposure could serve as a broadly effective form of support for children living in disadvantaged circumstances and create equity by “leveling the playing field” among disadvantaged and advantaged children. Finally, we identified implications of the current research for groups such as parents, teachers, and policymakers.

### 2.2 Procedure

#### 2.2.1 Phase 1: Key word search of major search engines

Given the exploratory nature of the project, and the fact that this literature is published across many disciplines, we employed a scoping review process aligning with that of Arksey and O’Malley (2005). It is our hope to advance theory and methods in this field by identifying gaps in the current body of research (Arksey and O’Malley, 2005; Colquhoun et al., 2014).

Three databases were searched in January 2025 for relevant, peer-reviewed research articles: EBSCO, PubMed, and Scopus. Four separate key word searches were conducted in each database using the following words or key word strings: *“equigenesis,” “equigenic,”* “mental health green space children socioeconomic,” and “psychology development green space children socioeconomic.” No limit was set on publication year during the searches.

Searches of the three databases using the key word strings resulted in 405 articles. After duplicate articles were removed within and between each of the databases, 154 articles remained. Titles and abstracts were screened for relevance to nature exposure’s effects on health outcomes, and 111 met that criteria.

Of those 111 articles, full texts were further evaluated for inclusion based on the following criteria:

- Directly examines the effects of greenspace on health outcomes, whether physical or psychological
- Written in English
- Empirical study or relevant literature review
- Not a prospective study
- Full text is accessible

After assessing the articles for inclusion, 92 articles remained. It should be noted that the inclusion criteria purposefully did not restrict age range, type of health outcome, or the inclusion of measures of advantage. This allowed for the detection of studies testing equigenesis within the context of the nature-health literature, revealing how many studies conducted tests for equigenesis versus those that only controlled for advantage or did not include advantage at all.

#### 2.2.2 Phase 2: Searching bibliographies and literature reviews

Of the articles selected—including peer-reviewed research literature review articles—bibliographies were examined for additional articles fitting the search criteria (“snowballing”; Greenhalgh and Peacock, 2005). That process identified an additional 51 articles. Thus, a total of 143 articles were included in the initial analysis for this review.

Our rationale for including literature reviews in our initial collection of studies was to fully understand the current state of the literature on nature exposure’s equigenic potential on physical and psychological health outcomes as well as to find relevant sources that may have been missed in the key word search. There were 20 (13.9%) literature reviews that presented relevant information on nature exposure’s effects on physical and psychological health. Of these reviews, 14 of the 20 discussed or mentioned greenspace potentially reducing health inequities (e.g., Alderton et al., 2019; Badland and Pearce, 2019; Fyfe-Johnson et al., 2021; Kuo et al., 2019; Markevych et al., 2017; Rigolon et al., 2021). While their findings contribute to the literature and what is known about equigenesis, after selecting relevant articles from their bibliographies, they were dropped from further analysis to construct a streamlined collection of empirical research studies examining nature exposure and health outcomes. Thus, 123 articles were included in the first stages of data extraction. See the Supplementary material for complete lists of the reviews and the 123 included empirical research studies.

#### 2.2.3 Phase 3: Evaluation of study variables and outcomes

To create an overview of the literature, key characteristics and elements of each study in the original dataset (*n* = 123) were charted. Such characteristics were year of publication, study location, health outcome, whether the study compared groups of advantage or not, and if so, whether the results of the study supported the equigenesis hypothesis or not. The first author evaluated all records, and the second author evaluated 25% of the articles to ensure consistent charting of the variables.

For location, studies with multiple countries were categorized under “Europe,” “Latin America,” “UK (Full & Partial),” and “Varied” depending on the locations of the included countries.

For health outcome, the measures for assessing health outcome were used as guidance for study categorization. For example, studies using the Strengths and Difficulties Questionnaire were categorized under “Socioemotional Functioning and Development,” even if studies referred to their dependent variable as “mental well-being.” Some studies used multiple methods to assess various outcomes and were thus included in multiple categories. See Supplementary Table 2 for the specifics of health outcome categorization.

In our evaluation, studies that compared groups of advantage were coded by their level of support for the equigenesis hypothesis. A study was coded as *supportive* when it had at least one statistically significant result showing disadvantaged groups benefiting more than advantaged groups from nature exposure, even if other results were non-significant. For specific examples of supportive evidence, see Table 1. The other categories for coding were *non-supportive* (advantaged groups benefited more), *conflicting* (the study found both supportive and non-supportive evidence), and *non-significant* (no statistically significant differences between groups).

**Table 1:** Forms of evidence supportive of the equigenic hypothesis.

Study design and analytic tests	Supportive evidence
Longitudinal, pre-post experimental	Narrowed gaps in health outcome between advantage groups over time, ideally due to disadvantaged groups improving more
Cross-sectional: effect modification	Significant interaction term favoring the disadvantaged group
Cross-sectional: stratified analysis by advantage status	Stronger associations between nature exposure and health outcome for the disadvantaged group; Significant positive associations for the disadvantaged group, non-significant associations for the advantaged group; Significant positive associations for the disadvantaged group, significant negative associations for the advantaged group(added semicolons after the first two paragraphs to distinguish items)

**Figure 1:**
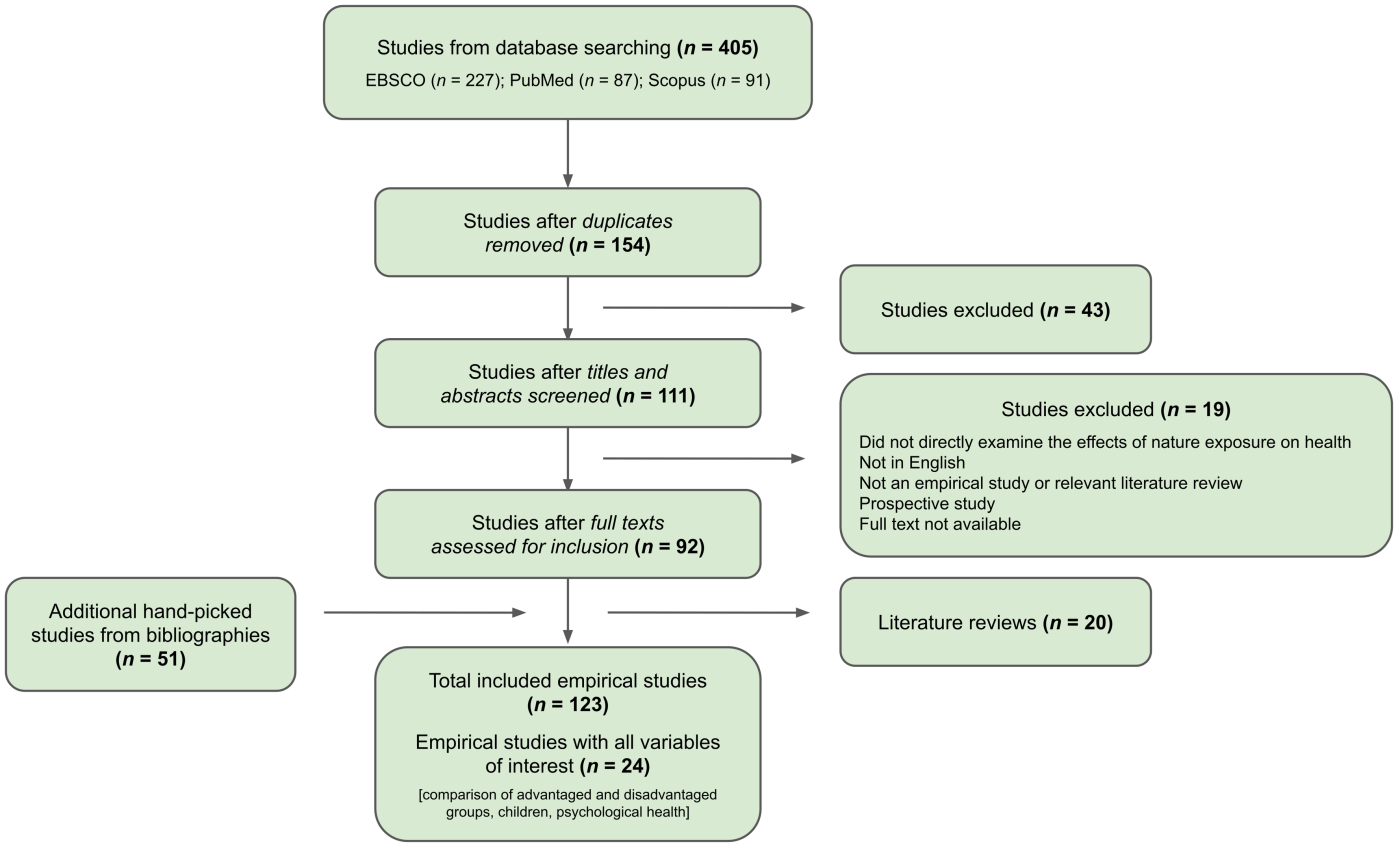
Flow chart of the literature search and selection process.

Of the 123 included articles, studies limited to children that also directly compared the health outcomes of advantage groups were then extracted for further examination. This refinement resulted in 30 articles. Of those, 24 articles included at least one psychological health outcome. Six articles, however, only measured physical health outcomes, and thus were eliminated from further analysis. Additional study characteristics were extracted and evaluated by both authors for each of the 24 studies along with the elements mentioned previously: age range, sample size, study design (cross-sectional, longitudinal, or experimental), measure for assessing psychological health outcomes, type of nature exposure, measure for assessing nature exposure, and whether the results of the study aligned with the equigenesis hypothesis or not. See Figure 1 for a flow chart of the literature search and selection process.

## 3 Results

### 3.1 Equigenesis for all ages and health outcomes

#### 3.1.1 Study characteristics

**Figure 2:**
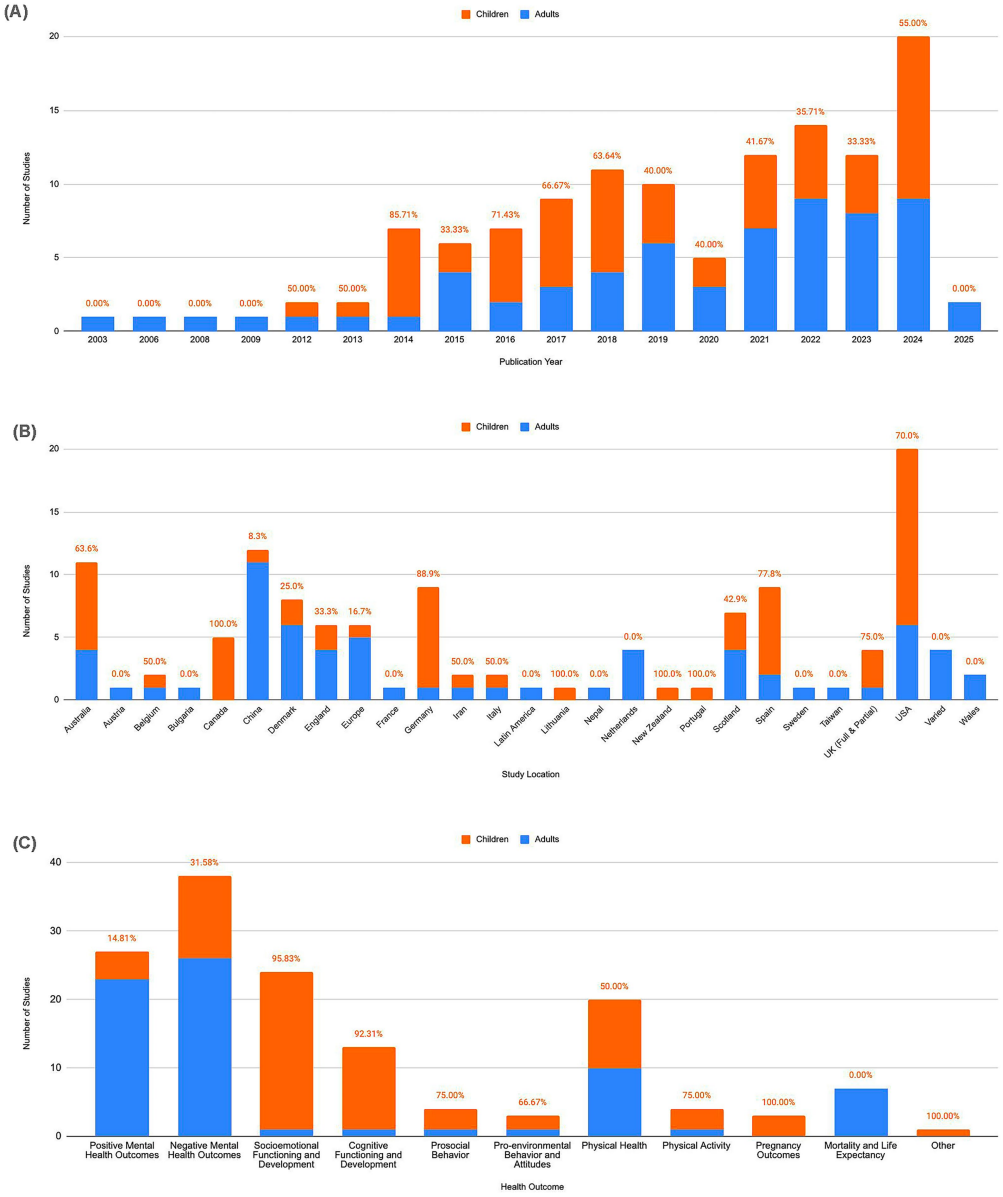
Study characteristics of all included studies (*n* = 123). **(A)** Studies organized by publication year. **(B)** Studies organized by country. **(C)** Studies organized by health outcome. The percentages on top of each bar indicate what percentage of studies within that bar were studies with children (orange). Panel **(C)** includes double counts, as some studies used multiple measures in their methods to assess various health outcomes.

123 studies were included in the first level of data selection with no restriction set on time of publication during the search. These studies were published between 2003 and 2025, with the number of studies published per year rising steadily over time—more than half of the studies (*n* = 65, 52.85%) were published between 2020 and 2025 (see Figure 2A).

Figure 2B shows the distribution of included studies by country. 108 (87.8%) studies took place in 23 distinct countries, with the United States, China, and Australia featuring the greatest numbers of studies. 15 (12.2%) studies collected data from multiple countries (i.e., columns labeled “Europe,” “Latin America,” etc.). By continent, the greatest number of studies were in Europe (i.e., all columns naming European countries; *n* = 65, 52.85%), followed by North America (i.e., “USA” and “Canada”; *n* = 25, 20.33%). The European studies had a relatively equal ratio of studies with children (*n* = 30, 46.15%) compared to studies with adults, while the North American studies contributed a greater proportion of studies with children (*n* = 19, 76%).

Various physical and psychological health outcomes have been explored throughout the literature in relation to nature exposure. Multiple studies explored more than one health outcome and thus used more than one measurement to quantify their health outcomes of interest (e.g., Fernandes et al., 2024). As evidenced by Figure 2C, the greatest number of studies analyzed mental health status (i.e., positive and negative mental health outcomes; *n* = 65), specifically in more psychiatric contexts (i.e., negative mental health outcomes; *n* = 38), although the majority (*n* = 49, 75.38%) of the mental health studies featured adult participants. The psychological health outcome with the greatest proportion of studies with child participants was socioemotional development, followed closely by cognitive development.

#### 3.1.2 Support for equigenesis

To understand if nature exposure has an equigenic effect on health outcomes for groups of differing levels of advantage, the outcomes for those groups must be compared directly rather than simply controlling for advantage variables. Of the included studies, four (3.25%) studies did not include an advantage variable, and 55 (44.72%) measured advantage status (income, highest level of education, etc.) but not for the purpose of comparing advantage groups. Thus, those studies have been excluded from further discussion in this paper. The remaining 64 (52.03%) studies did compare advantage groups in relation to nature exposure and health outcomes, thus testing for equigenesis.

**Figure 3:**
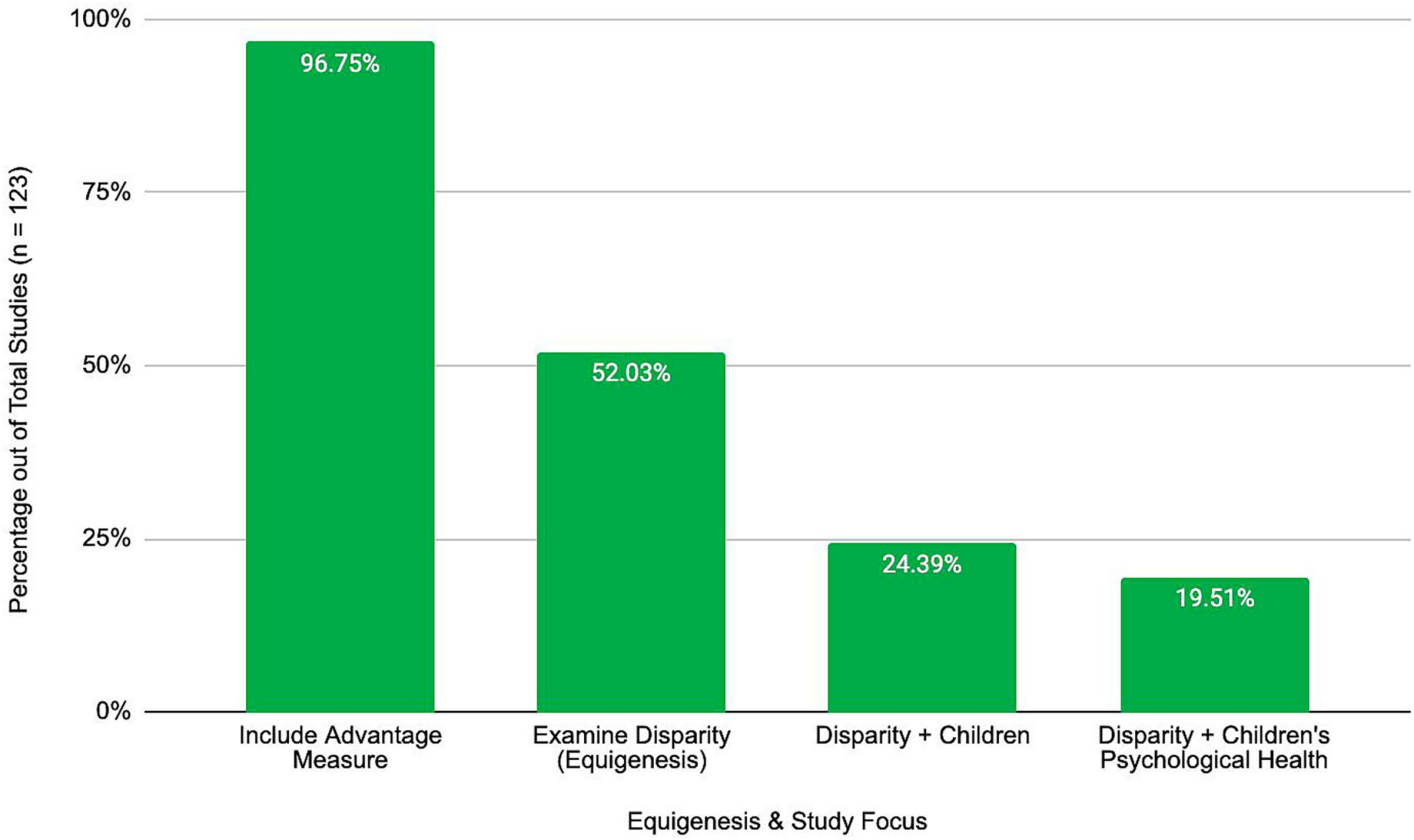
A visualization of the state of the literature in relation to studies of equigenesis in children’s psychological health. Only 19.51% of the literature reviewed featured all three variables of interest.

It is important to note only 30 (24.39%) of the 123 articles examined equigenesis specifically in children, and only 24 (19.51%) examined equigenesis in relation to children’s psychological health (see Figure 3).

**Figure 4:**
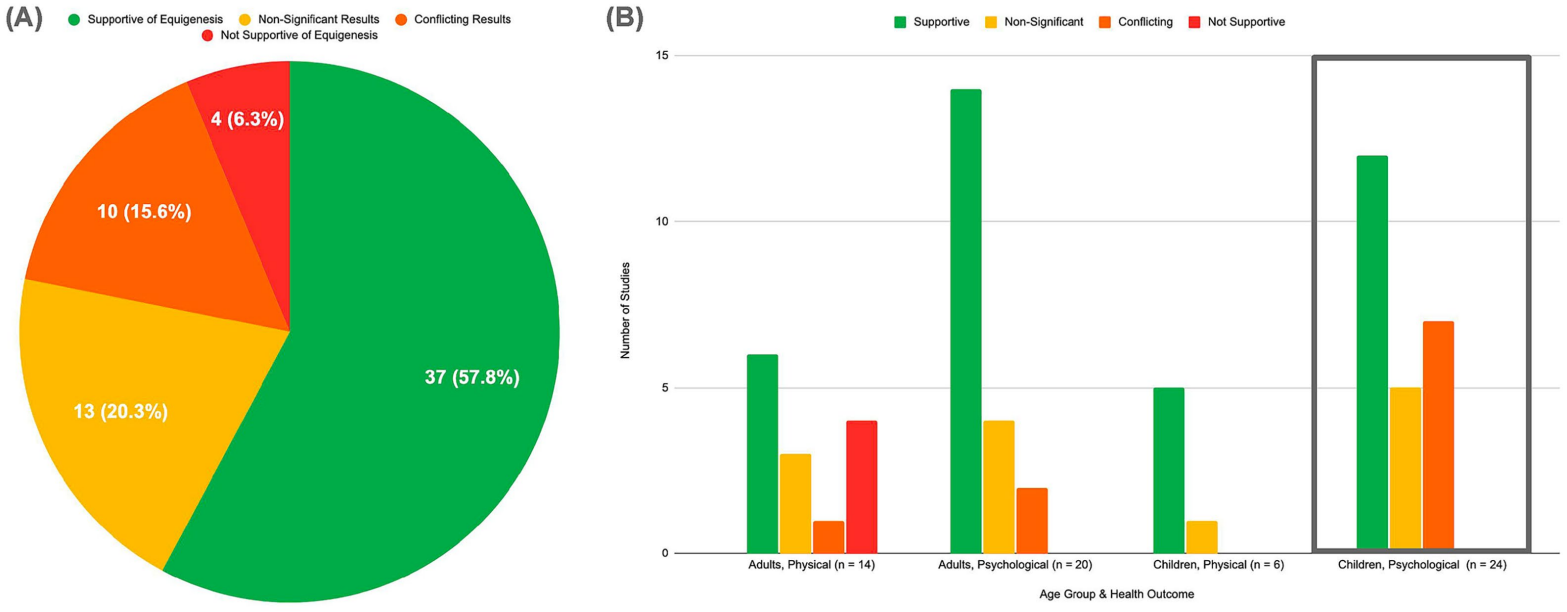
Support from the literature for nature exposure’s equigenic effects on health outcomes for all ages (*n* = 64). **(A)** Total breakdown of support for equigenesis by study. **(B)** Breakdown of support for equigenesis by age group and health outcome type.

Of the 64 studies with adults or children directly comparing groups of differing advantage, 37 (57.81%) found supportive evidence for nature exposure’s equigenic effects on health outcomes (see Figure 4A). Although, three of those studies demonstrated narrowed inequalities partially due to the advantaged group displaying negative psychological outcomes in association with nature exposure, which is obviously not desirable (Balseviciene et al., 2014; Browning et al., 2022; Pérez-del-Pulgar et al., 2021). Thirteen (20.31%) studies were coded as non-significant; they did not indicate any statistically significant differential relationships between advantage groups for positive outcomes from nature exposure. Ten (15.63%) studies revealed conflicting findings; nature exposure was linked with significant improvements on some measures with the disadvantaged group benefiting more, but the same study also found a significant association on a different outcome measure in which the advantaged group benefited more. Different associations were detected between different measures of nature exposure, advantage (e.g., family income vs. parental education), and health outcome, which will be further explored in the discussion. Four (6.25%) of the studies, which all measured physical health outcomes in adults, reported significantly *widened* disparities between advantage groups, although some of those pointed to low statistical power as a potential explanation (Cole et al., 2019; Elliott et al., 2023; Plans-Beriso et al., 2024; Wei et al., 2024). Figure 4B presents the studies testing for equigenesis subdivided by age and health outcome, revealing that the majority of studies across all groups report supportive findings.

### 3.2 Equigenesis for children’s psychological health

#### 3.2.1 Study characteristics

Twenty-four empirical studies specifically examined equigenic outcomes for children’s psychological health. Their descriptive characteristics are outlined below in Table 2. Ten (41.67%) studies assessed children specifically in early childhood (3–7 years), whether as the complete sample in a cross-sectional design or the initial sample in a longitudinal design. The publication years of the studies (2014–2024) are skewed toward more recent years, and the greatest number of studies took place in the United States (*n* = 7, 29.17%). Psychological health constructs evaluated included anxiety, behavioral difficulties, psychological and behavioral disorders, academic achievement in math and reading, cognitive functioning, and prosocial and pro-environmental behaviors. Thematically, the most-researched psychological health variables for equigenic studies with children were socioemotional functioning (*n* = 12), followed by cognitive functioning (*n* = 6). In terms of methods, a variety of measures were used across studies to quantify nature exposure. The most used measure of nature exposure was the Normalized Difference Vegetation Index (*n* = 9). The studies also employed large sample sizes, with 18 (75%) studies featuring sample sizes of over 500. See Supplementary Figure 1 for the distributions of these study characteristics.

**Table 2:** Studies that compare advantaged children to disadvantaged children in terms of psychological health in relation to nature exposure (*n* = 24).

Study	Study location	Age range (years)/grade level	Sample size	Study design	Psychological health outcome	Psycho-logical health measure	Type of nature exposure	Nature exposure measure	Supports equigenesis?
Balseviciene et al. (2014)	Kaunas, Lithuania	4–6	1,468	Cross-sectional	Emotional and behavioral development	SDQ	Greenspace quantity, proximity to parks	NDVI around residential address (300 m buffer), proximity to nearest park	Yes
Bernardo et al. (2021)	Lisbon, Portugal	Grade 3	75	Quasi-experimental	Sustained and selective attention and working memory	Bells Test, Digit Span Test from Wechsler Intelligence Scale for Children	Indoor greenery, environmental education program	Classroom greenery and vegetable growing activity	Non-significant results*
Bolanis et al. (2024)	Quebec, Canada	10 ➔ 15–17	742	Longitudinal	Mental health (inattention, hyperactivity/impulsivity, conduct, depression, anxiety, suicidal ideation)	Mental Health and Social Inadaptation Questionnaire	Greenspace quantity	NDVI around residential address (250 m, 500 m, and 1,000 m buffers)	Non-significant results
Bølling et al. (2019)	Denmark	9–13	631	Quasi-experimental	Psychosocial well-being	SDQ	Nature-based learning	“Education Outside the Classroom” (teaching mainly in natural settings)	Yes
Caryl et al. (2024)	Scotland	10–11	640	Cross-sectional	Mental well-being	SDQ	Use of greenspaces	Intersection between global positioning system (GPS) location and natural landcover as determined by landcover data, “passive” versus “active” use	Yes
Dadvand et al. (2019)	Iran	10–18	10,856	Cross-sectional	Self-satisfaction and social contacts	Questionnaire designed for study	Use of greenspaces	Parent- and child-reported data on time spent in public greenspaces	Conflicting results
de la Osa et al. (2024)	Barcelona, Spain	3 ➔ 10–11	539	Longitudinal	Anxiety	CBCL, SCAS-P	Greenspace quantity, proximity to greenspace	NDVI, MSAVI, and VCF around residential address and school (50 m, 100 m, 300 m, and 500 m buffers), DCG	Yes
Ernst and Stelley (2024)	Minnesota, USA	3–5	115	Quasi-experimental	Self-regulation (hot and cold executive control, attention/impulse control)	Preschool Self-Regulation Assessment (PSRA), PSRA Assessor Report	Nature-based learning and play (indoor nature content, outdoor greenspace)	Nature-based practices in preschool	Yes
Ernst et al. (2022)	Minnesota, USA	3–5	147	Quasi-experimental	Executive function	Minnesota Executive Function Scale	Nature-based learning and play (indoor nature content, outdoor greenspace)	Nature-based practices in preschool	Yes
Ezpeleta et al. (2022)	Barcelona, Spain	9–10	378	Cross-sectional	Obsessive-compulsive behaviors	SCAS-P	Greenspace quantity, proximity to greenspace	NDVI, MSAVI, and VCF around residential address and school (50 m, 100 m, 300 m, and 500 m buffers), DCG	Conflicting results
Fernandes et al. (2024)	8 European countries (Netherlands, UK, Denmark, France, Spain, Lithuania, Norway, Italy)	3–12	35,407	Cross-sectional	Neurodevelopmental outcomes (non-verbal intelligence, internalizing and externalizing problems, attention deficit hyperactivity disorder)	Various neuropsychological assessments across cohorts	Greenspace quantity, proximity to greenspace	NDVI around residential address (300 m buffer), DCG	Non-significant results
Flouri et al. (2014)	UK	3–7	6,384	Longitudinal	Emotional and behavioral problems	SDQ	Greenspace availability	Neighborhood LULC data	Yes
Hartley et al. (2023)	North Carolina, USA	7–11	644	Experimental (pre-post design)	Pro-environmental behavior	Questionnaire designed for study	Environmental education program	Environmental education curriculum about marine debris (not always outdoors)	Yes
Hazlehurst et al. (2024)	Tennessee, USA	4–6	943	Cross-sectional	Internalizing and externalizing behavior	CBCL	Greenspace quantity, proximity to parks	NDVI and tree cover data around residential address (300 m buffer), proximity to nearest park	Non-significant results*
Jimenez et al. (2022)	Massachu-setts, USA	3.1 ➔ 7.8 (medians)	857	Longitudinal	Cognitive functioning	Cognitive assessments	Greenspace quantity	NDVI around residential address (90 and 270 m buffers)	Conflicting results
Kuo et al. (2018)	Illinois, USA	Grade 3	318 schools	Cross-sectional	Academic performance (reading, mathematics)	Standardized test scores	Greenspace quantity	Tree and grass/shrub cover data for neighborhood, school, and neighborhood-and-school areas	Yes
McCrorie et al. (2021)	Scotland	10–11	774	Cross-sectional	Social, emotional, and behavioral well-being	SDQ	Greenspace availability	LULC [natural space] data around residential address (100 m buffer), private garden access	Conflicting results
McEachan et al. (2018)	Bradford, England, UK	4–5	2,594	Cross-sectional	Mental well-being	SDQ	Quantity, use of, and satisfaction with greenspaces	NDVI around residential address (100 m, 300 m, and 500 m buffers), survey questions completed by parents regarding weekly use of public greenspaces and satisfaction with quality of public greenspaces	Yes
Nigg et al. (2022)	Germany	4–17	2,843	Cross-sectional	Mental health	SDQ	Greenspace availability	LULC data around residential address (different buffer types and different buffer sizes based on type)	Conflicting results
Pérez-del-Pulgar et al. (2021)	Barcelona, Spain	0–12	151,110	Cross-sectional	Psychological disorder prevalence	Diagnoses	Proximity to residential outdoor play spaces	Census tract intersections with buffers around outdoor play spaces	Yes
Putra et al. (2021)	Australia	4–5 ➔ 14–15	4,969	Longitudinal	Prosocial behavior	SDQ	Greenspace quality	Caregiver-report of availability of high-quality neighborhood greenspaces	Conflicting results
Richardson et al. (2017)	Scotland	4–6	2,909	Longitudinal	Social, emotional, and behavioral well-being	SDQ	Greenspace availability	LULC data around residential address (500 m buffer), private garden access	Conflicting results
Sivarajah et al. (2018)	Toronto, Canada	Grades 3 and 6	387 schools	Cross-sectional	Academic performance (reading, writing, mathematics)	Academic data by school	Greenspace quantity and diversity	Tree cover and species composition data by school	Yes
Younan et al. (2016)	California, USA	9–18	1,287	Longitudinal	Aggression	CBCL	Greenspace quantity	NDVI around residential address (250 m, 350 m, 500 m, and 1,000 m buffers)	Non-significant results

CBCL = Child Behavior Checklist; DCG = Distance to Closest Greenspace; LULC = land use/land cover data; MSAVI = Modified Soil Adjusted Vegetation Index; NDVI = Normalized Difference Vegetation Index; SCAS-P = Spence Children’s Anxiety Scale, Parent Version; SDQ = Strengths and Difficulties Questionnaire; VCF = Vegetation Continuous Fields. Asterisks indicate associations trending in the hypothesized direction of equigenesis but did not achieve statistical significance.

#### 3.2.2 Support for equigenesis

Of the literature on children’s psychological health comparing groups of differing advantage, 50% (12 out of 24) found that exposure to nature was associated with greater psychological benefits in children living with disadvantage when compared to their more advantaged peers (see Figure 4B). More specifically, the studies suggested that in comparison to children living with more advantage, children living with greater disadvantages displayed stronger outcomes associated with nature exposure including lower levels of anxiety, behavioral difficulties, and psychological and behavioral disorders as well as improved reading and math skills, cognitive functioning, and prosocial and pro-environmental behaviors. Including seven studies that reported conflicting findings, 79.17% of the studies revealed some evidence for equigenesis. None of the studies found evidence against the equigenesis hypothesis for children’s psychological health—widening gaps or stronger outcomes for advantaged children—but five studies had non-significant findings. Next, we will explore noteworthy themes in the literature and possible mechanisms underlying the potential equigenic relationship between nature exposure and children’s psychological health.

## 4 Discussion

### 4.1 Overall state of the literature – Supportive and conflicting findings

This review of the literature reveals a growing body of research in support of the hypothesis that nature exposure can have equigenic effects on physical and psychological health outcomes for adults and children. However, only 20% examined equigenesis in conjunction with children’s psychological health, demonstrating that little work has explored the potential for nature exposure as an equigenic influence for children living with disadvantage. Within the existing equigenic literature on children’s psychological health, many—19 out of 24, including studies with conflicting findings—demonstrate at least one measure indicating an equigenic effect on the advantage-disadvantage gap in children’s mental health and psychological development outcomes, either through comparing outcomes over time or demonstrating larger effect sizes and stronger associations (see Table 2).

However, some studies in the children’s psychological health literature did not demonstrate a gap reduction. In those studies, all groups had improved outcomes; in other words, the disadvantaged group did not show greater gains than the advantaged group (e.g., Fernandes et al., 2024; Hazlehurst et al., 2024; Younan et al., 2016). These non-significant findings have the potential to be viewed as evidence against the equigenesis hypothesis; however, reasons such as low statistical power or problematic measures may have led to the lack of statistical significance rather than nature exposure working equally on both groups (e.g., Bernardo et al., 2021; Bolanis et al., 2024). There were also a few studies that demonstrated a gap reduction, but partly due to advantaged children’s outcomes declining in association with nature exposure, which is not a desired means of achieving equigenesis in population health (Balseviciene et al., 2014; Browning et al., 2022; Mitchell, 2013; Pérez-del-Pulgar et al., 2021). Other studies had mixed findings, depending on the measures employed (see Table 2). This variation in differences between advantage groups occurred across measures of advantage, nature exposure, and outcomes with children’s psychological health. Regarding variations due to measures of advantage employed, one study found that the gap in children’s verbal intelligence was narrowed in early childhood when analyses were stratified by neighborhood socioeconomic status but widened when stratified by parental education (Jimenez et al., 2022). This inconsistency has also been seen with adults; inequalities in emotional well-being were narrowed when moderated by participants’ income level but widened when moderated by participants’ education level (Guo et al., 2025).

Regarding variations due to measures of nature exposure, three studies spanning middle childhood that employed more than one measure of nature exposure revealed different equigenic findings depending on the measure. One study exploring the conceptualization and methodology behind nature exposure measures investigated how different scales and operationalizations of nature affect the relationship between greenspace and children’s psychological health and found heterogenous results by socioeconomic status based on buffer size, buffer type, and the definition of nature used (Nigg et al., 2022). As for more specific nature locations, a study found that smaller distances from greenspaces at home were associated with less obsessive-compulsive behaviors in children from lower socioeconomic levels, but the quantity of greenness at school was associated with less obsessive-compulsive behaviors in children from higher socioeconomic levels (Ezpeleta et al., 2022). Another study demonstrated that while household income moderated the associations between neighborhood greenspace and prosocial behavior equigenically, the associations were flipped when considering nature exposure to be access to a private garden, with advantaged children benefitting the most (McCrorie et al., 2021). In addition to those three studies, a study that employed both longitudinal (ages 4–15) and cross-sectional designs found longitudinal evidence for nature exposure narrowing gaps in prosocial behavior, but its cross-sectional analysis revealed widened gaps, with only advantaged groups demonstrating benefit (Putra et al., 2021).

Different psychological health outcome measures within studies have also yielded mixed findings. A longitudinal study found that greater percentages of natural space around a child’s home were associated with fewer peer problems in children from lower education households (narrowed gap) but better prosocial behavior in children from higher education households (wider gap) (Richardson et al., 2017). This inconsistency has also been reported in a study of adults measuring physical health, specifically mortality outcomes (Moran et al., 2021).

### 4.2 Themes in the literature regarding nature exposure

Studies of the equigenic relationship between nature exposure and children’s psychological health indicate aspects of nature that may make it more supportive: the quality of nature, frequency of use, contextual setting, and early childhood exposure.

#### 4.2.1 Quality of nature matters

The quality of greenspaces may be more important than the quantity available in relation to nature exposure’s equigenic effects on psychological health. This has been demonstrated in several studies with adults (e.g., Allard-Poesi and Massu, 2023; Fian et al., 2024; Wheeler et al., 2015). For example, Wang et al. (2022) found that greenspace quality, but not greenspace quantity, was associated with narrowed socioeconomic inequalities in depression and anxiety. Wang et al. (2021) proposed that greenspace quantity and quality operate via different mechanisms when benefitting psychological health. They argued that two potential pathways related to greenspace quality (restoring capacities, such as attention, and building capacities) may specifically benefit disadvantaged populations more than the mechanisms related to greenspace quantity (reducing environmental harms such as pollution).

Fewer studies with children have explored this. Two studies with children employed measures of greenspace quality, albeit limited to self-report, and tested for equigenesis in relation to psychological health. However, the authors of both studies claimed that self-report—in these studies, caregiver perceptions—can be used as a proxy for measuring nature quality due to quality predicting satisfaction and satisfaction’s influence on subsequent use. McEachan et al. (2018) found that reported greenspace satisfaction was associated with fewer internalizing and total behavioral difficulties as well as greater prosocial behavior in ethnically minoritized children ages 4–5. However, no significant moderations were found by socioeconomic status. Conversely, Putra et al. (2021) found in their longitudinal analysis, spanning ages 4–15, that satisfaction with greenspace may reduce socioeconomic inequalities related to prosocial behavior over time. In light of these results, future research should be intentional about measuring greenspace quality in addition to quantity.

#### 4.2.2 Frequency of nature use matters

Time spent in nature—number of visits over a period of time or quantity of time measured in minutes or hours—may predict equigenic benefits for children facing disadvantage, although there are some mixed results. One recent study used global positioning system (GPS) tracking and found stronger associations for the relationship between direct greenspace use and mental well-being for children from lower income households (Caryl et al., 2024). Another study of adolescents found that parental education modified the positive association between average hours per week spent in greenspaces and the number of and quantity of time spent with social contacts (Dadvand et al., 2019). However, this finding does not directly support the equigenesis hypothesis; youth with the least educated parents and youth with the most educated parents both had slightly stronger associations than the mid-level education group. That same study also did not find that parental education modified the association between time in greenspaces and a measure of self-satisfaction. In a different study, no evidence was found for frequency of nature use having an impact on equigenic effects; the study detected moderation by ethnic group only in relation to satisfaction with greenspace, not time spent in greenspace in early childhood (McEachan et al., 2018).

#### 4.2.3 Context of nature – Home vs. school

Children spend many hours per week at school, especially those receiving before and after care. Additionally, schools bring together children from diverse backgrounds. Thus, nature experiences at school have potential for fostering equigenic effects on children’s psychological health, perhaps more so than residential greenspaces. Three studies directly compared the effects of residential greenspace to school greenspace. In one study, school tree cover was related to third graders’ academic performance and was moderated by level of advantage; neighborhood tree cover was also related to academic performance but was not moderated by level of advantage (Kuo et al., 2018). In another study, greenspace surrounding school was associated with lower anxiety scores in children with low parental education compared to residential greenspace (de la Osa et al., 2024). However, as mentioned previously, Ezpeleta et al. (2022) found that school greenspace was associated with less obsessive-compulsive behaviors and was particularly beneficial for higher income children, thus not supporting the equigenesis hypothesis.

Other studies, while not comparing to residential greenspace, also exemplify the effects of school greenspace and nature-based educational practices on children’s psychological health. One study found that tree cover at school was significantly correlated with higher academic performance (sixth grade math, reading, and writing scores) for socioeconomically challenged schools while tree cover was non-significant for schools with lower levels of challenge (Sivarajah et al., 2018). Three studies involving employing nature-based teaching found that children from lower socioeconomic backgrounds benefitted more than their peers from higher socioeconomic backgrounds on at least one measure of psychological health (Bølling et al., 2019; Ernst et al., 2022; Ernst and Stelley, 2024). The same pattern was seen in a study employing environmental education focused on marine debris, however, it is unclear whether students were experiencing nature directly when receiving lessons (Hartley et al., 2023). One study of indoor classrooms with added greenery found pre-post improvements in working memory and sustained and selective attention in both low- and high-income groups; however, there was not a significant moderating effect of income, thus not demonstrating equigenic effects (Bernardo et al., 2021).

#### 4.2.4 Early childhood a “critical period” for equigenic effects?

Two longitudinal studies in the final dataset suggest that there may be a specific window in early childhood during which residential nature yields the greatest equigenic effects. One study found that the association between fewer emotional problems and residential greenspace for boys from backgrounds of socioeconomic disadvantage was no longer observable after age five (Flouri et al., 2014). Another detected associations between social, emotional, and behavioral outcomes and neighborhood greenspace that were moderated by household education, and children from lower education households without private garden access had worse outcomes. However, no changes were detected between the baseline of age four and age six for children from lower education households, suggesting that greenspace benefits occurred before age four (Richardson et al., 2017). Thus, nature exposure earlier in childhood may prove more beneficial for reducing inequalities. However, this phenomenon requires further examination, including tests of earlier ages and nature exposure at school. The inclusion of nature at school may dilute detectable benefits of residential nature, explaining the lack of findings for children after entering school (Flouri et al., 2014). The findings of Kuo et al. (2018) support this concept, which were discussed in the previous section.

### 4.3 Mechanisms underlying the equigenic relationship between nature and children’s psychological health

Long before studies looking into equigenic effects of nature exposure, researchers have been examining potential mechanisms behind the nature-health relationship to understand why and how nature provides health benefits. Regarding psychological health, the Attention Restoration Theory (ART) and Stress Reduction Theory (SRT) are often cited as means of building attentional capacities and relieving stress through nature exposure (Kaplan, 1995; Ulrich et al., 1991). Additionally, research has posited other mechanisms that mediate the relationship between nature exposure and psychological health, such as reduced exposure to harmful environmental factors and increased physical activity (e.g., Dzhambov et al., 2018; Markevych et al., 2017). For children specifically, nature has been shown, for example, to encourage free play and exploratory thinking, and hands-on practices in nature-based education support learning (for review see Kuo et al., 2019). Improvements in psychological outcomes in association with nature exposure may be at least partially explained by some of these mechanisms.

**Figure 5:**
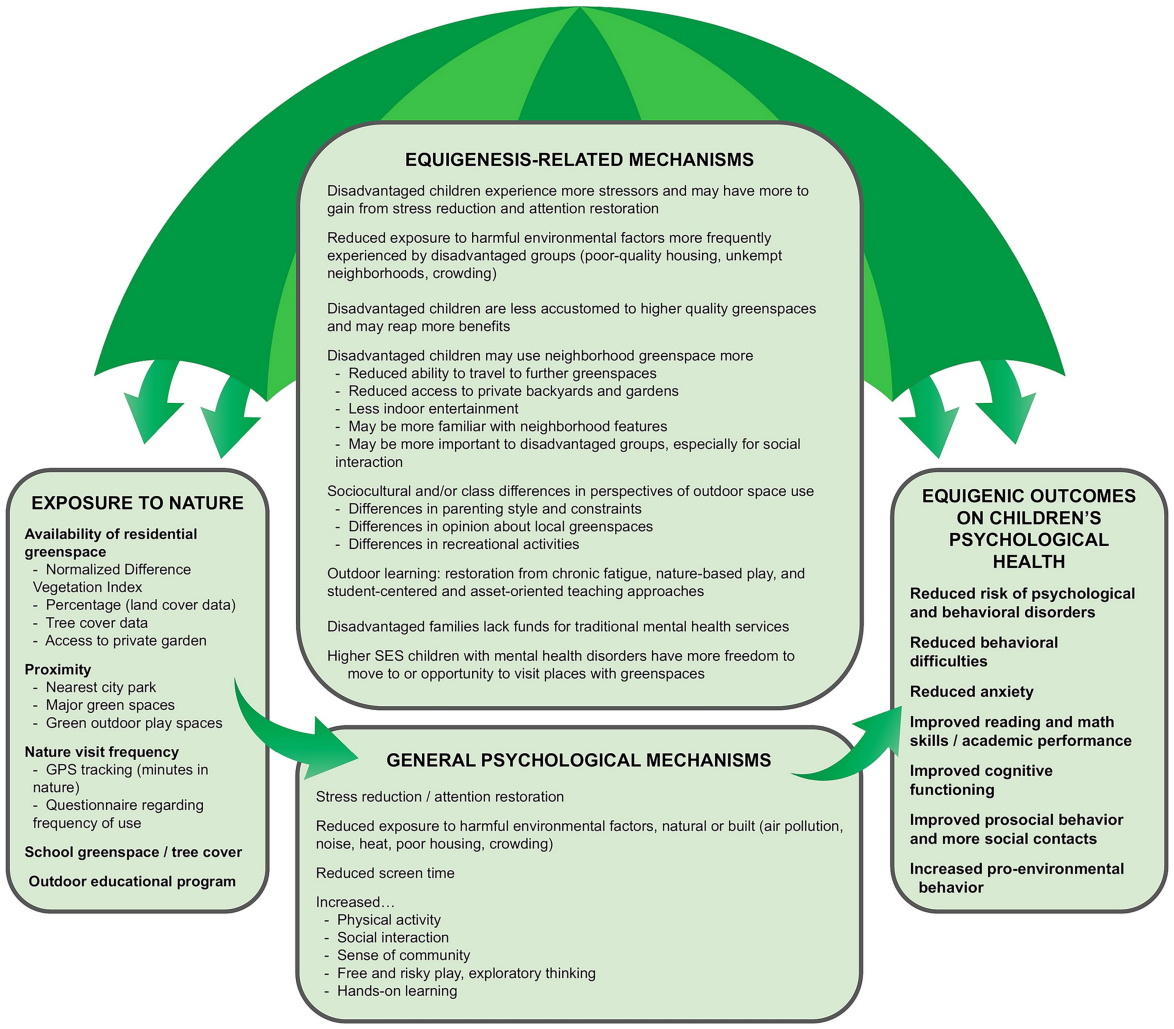
Our proposed framework of the “umbrella” of potential equigenesis-related mechanisms acting on the nature-health pathway, specifically in the context of children’s psychological health. Not every form of nature exposure listed has been associated with every single psychological outcome listed. The purpose of this figure is to pool all of the variables cited in the literature into a comprehensive diagram of the evidence for equigenesis so far.

We propose that nature exposure’s equigenic effects on children’s psychological health may be operating through multiple potential mechanisms simultaneously. Rather than a single mechanism at work, such as stress reduction, it is likely that multiple mechanisms pertinent to the differential experiences of advantage levels are simultaneously involved, influencing the effectiveness of both the independent variable (nature exposure) and its related mechanisms on the dependent variables (psychological health outcomes). These equigenesis-related mechanisms may affect how much benefit is received from nature exposure due to different levels of exposure to nature influenced by advantage status. They may also affect the influence of the generally accepted mechanisms explaining why and how nature exposure benefits psychological health (e.g., ART and SRT) through varied susceptibility by advantage level. Such mechanisms explain why one advantage group might benefit *more* than another in the relationship between nature exposure and dependent variables, resulting in a more “level playing field” between advantaged and disadvantaged children. A visualization of this concept is an “umbrella” of overarching mechanisms acting on the entire nature-health pathway (see Figure 5). In the following paragraphs, we explore three broad potential mechanisms that may be helpful in interpreting equigenic outcomes in relation to nature exposure.

#### 4.3.1 Disadvantaged children possess greater psychological needs due to experiencing more stressors

It has been proposed that children facing disadvantage may have more room for improvement in psychological health than their advantaged peers, which is the main tenet behind the equigenesis hypothesis and may explain research findings showing disadvantaged children reaping greater benefits from nature exposure (Balseviciene et al., 2014; Businelle et al., 2014; Caryl et al., 2024; Kuo et al., 2018; Mitchell et al., 2015; Putra et al., 2021; Yoshikawa et al., 2012). Children with disadvantage commonly experience chronic stressors including parental stress, material hardships, and poor neighborhood conditions, which have negative consequences for psychological health (Chaudry and Wimer, 2016; Johnson et al., 2016). Thus, children living in disadvantaged circumstances may have greater ongoing needs for attention restoration and stress reduction. If advantaged children are already experiencing better psychological health, lack of findings may be an indication of a “ceiling effect,” demonstrating less room for improvement and less of a need for psychological support from nature exposure. Therefore, their disadvantaged peers can “catch up,” displaying greater gains in psychological health.

The presence of nearby nature also may be more impactful for disadvantaged than advantaged children in that nature can reduce exposure to environmental factors that may exacerbate psychological stress (Markevych et al., 2017; McCrorie et al., 2021; Pérez-del-Pulgar et al., 2021). Harmful environmental exposures such as air pollution or noise are more likely to be experienced by disadvantaged populations due to impoverished neighborhoods, but the impact of those conditions can be buffered by the implementation of natural features (Markevych et al., 2017; Morenoff and Lynch, 2004). Furthermore, visiting nearby greenspaces may provide a break from conditions of poor-quality housing, overcrowding, and violence experienced by those in disadvantaged neighborhoods.

Greenspace *quality* may also explain why children with disadvantage benefit more than children with advantage, who may be more accustomed to high quality greenspaces due to continual access. In contrast, children with disadvantage are more likely to have lower quality natural spaces in their neighborhoods (Chenyang et al., 2022). Due to this imbalance, children with disadvantage may reap more psychological benefits when exposed to high quality greenspaces, particularly if high quality greenspaces foster more benefits in comparison to low quality greenspace, such as physical activity and social cohesion (Wang et al., 2022). It is even more imperative that disadvantaged children have access to high quality greenspace especially if greenspace quality works through mechanisms such as attention restoration and stress reduction, which more commonly need support in disadvantaged populations, as mentioned previously (Wang et al., 2022).

#### 4.3.2 Disadvantaged children may experience neighborhood greenspace more

Perhaps equigenic findings in studies of residential nature may also be the result of disadvantaged children simply using their local greenspaces more than advantaged children. This may be due to a variety of factors. For example, due to poor health and fewer financial resources, disadvantaged populations are less likely to be able to travel to further greenspaces and are thus more likely to primarily use their neighborhood greenspaces (Balseviciene et al., 2014; de la Osa et al., 2024; Pérez-del-Pulgar et al., 2021; Wang et al., 2022). Given that children are generally dependent upon parents for transportation, children in disadvantaged households are more likely to be limited to resources they can access independent of their family. Thus, they are more familiar with their neighborhood resources and more likely to visit nearby greenspaces (Putra et al., 2021). Children in disadvantaged households are also less likely to have access to private backyards and gardens as well as indoor entertainment, which further encourages use of neighborhood greenspaces (Dadvand et al., 2019; McCrorie et al., 2021; Pérez-del-Pulgar et al., 2021).

Some studies suggest that disadvantaged groups may use local greenspace more than advantaged children due to sociocultural differences less directly related to financial strain (McCrorie et al., 2021; Pérez-del-Pulgar et al., 2021). Research has demonstrated that middle-to-upper class parenting styles, time management, and concerns about safety may hinder advantaged children from using local greenspace (Pérez-del-Pulgar et al., 2021). Conversely, as disadvantaged populations may tend to use their local greenspaces more often, they may develop a sense of pride in their neighborhoods’ features, which may in turn further increase frequency of use (Pérez-del-Pulgar et al., 2021). Advantaged children also have greater access to and are more likely to be enrolled in recreational activities farther from home or in non-greenspaces (e.g., recreational centers), while children living in disadvantaged circumstances may be more reliant on local spaces such as greenspaces for their sense of community (Simpkins et al., 2005). If equigenesis has been mainly studied in the context of near-home nature rather than testing other types of nature experiences, then perhaps equigenic effects are detected due to disadvantaged children experiencing that form of nature more than advantaged children.

On the other hand, disadvantaged populations often face personal, social, and physical barriers that may make them *less* likely to use their local greenspaces (Chenyang et al., 2022). Concerns about safety or cleanliness as well as lack of high-quality greenspaces—more common in disadvantaged neighborhoods—may reduce attraction and dissuade use (Badland and Pearce, 2019; Chenyang et al., 2022; McEachan et al., 2018; Putra et al., 2021; Richardson et al., 2017). Disadvantaged neighborhoods also often have a lack of organized outdoor activities, and parents with lower levels of education may have more time-consuming occupations that keep them from being able to chaperone their children outside, limiting use (Chenyang et al., 2022; Dadvand et al., 2019; Jimenez et al., 2022). The presence of these barriers suggests that equigenic findings are more likely fostered by the psychological mechanisms discussed previously rather than attributable to disadvantaged children having more frequent exposure to neighborhood greenspaces.

#### 4.3.3 Nature experiences at school may support disadvantaged children more than traditional academic settings

Mechanisms that may explain why disadvantaged children may benefit more from greenspace at school include restoration from chronic fatigue, opportunities for nature-based play, and student- and asset-centered teaching approaches. A study of school tree cover in relation to academic performance argued that the restorative effects of greenspace may be more salient in disadvantaged children given that they are more likely to experience chronic mental fatigue resulting from chronically difficult conditions at home (Kuo et al., 2018). Additionally, preschools in impoverished areas tend to have fewer nature-based play opportunities in the daily schedule, relying more on teacher-directed instruction than higher income schools (Ernst et al., 2022). Thus, the inclusion of nature-based play and its benefits in the curriculums of disadvantaged schools may help boost disadvantaged children to the level of their more advantaged peers who already experience the benefits of nature-based play (Kuo et al., 2019). Lastly, nature-based education may be more helpful for children facing disadvantage due to its nontraditional methods of instruction (Hartley et al., 2023; Kuo et al., 2019). As discussed by Hartley et al. (2023), the student- and asset-centered perspectives that are often associated with nature-based education allow for the integration of various cultural perspectives, highlighting agency and diversity in ways that can help children facing disadvantage perform better in academics than they would in more traditional academic settings. However, given that disadvantaged schools are often less green and more likely to have fewer recess breaks (Kuo et al., 2018), the disproportionate benefits sometimes detected for disadvantaged children hint that other mechanisms may be involved beyond disadvantaged children simply experiencing more greenspace when at school.

#### 4.3.4 Additional potential mechanisms

Other variables unrelated to psychological capacities and frequency of use might partially explain an equigenic effect in the relationship between nature exposure and psychological health. For example, children from disadvantaged circumstances may demonstrate greater psychological benefits in relation to nature exposure than their more advantaged peers because it is a more significant source of their on-going support whereas their advantaged peers may also be receiving purchased support such as fee-based mental health services thus diluting the impact of nature exposures (Triguero-Mas et al., 2017; Wang et al., 2022). Children in disadvantaged households also lack housing mobility whereas advantaged families seeking to support a child in poor psychological health may move closer to facilities, including greenspaces, that support well-being (Pérez-del-Pulgar et al., 2021). This would explain the tendency for advantaged children as a group to experience less psychological gains over time.

### 4.4 Limitations

There are some limitations to this literature review. The key word combinations employed in the database searches may not have revealed all relevant studies. However, a “snowballing” technique (Greenhalgh and Peacock, 2005) of searching bibliographies of studies found was employed to find additional relevant studies. Only studies published in English with the full text available were included in this review, which may have inadvertently excluded relevant findings.

Though “vote counting” to categorize studies and summarize findings is common in literature reviews, we acknowledge the weaknesses in this approach (Cumpston et al., 2023). We employed vote counting to categorize the studies’ findings as supportive of the equigenesis hypothesis or other. For example, categorizing as supportive based on statistical significance excludes underpowered studies from being counted, which may lead to underestimating the evidence for nature’s equigenic effects as well as only discussing statistically significant findings (Cumpston et al., 2023; McKenzie and Brennan, 2019). However, the studies in our dataset often had very large sample sizes, which combats inadequate statistical power. Conversely, publication bias towards studies with statistically significant findings may inflate the number of studies showing support, overestimating the quantity of evidence for nature’s equigenic effects.

Admittedly, this literature review has a risk of bias. Hand-picking studies from bibliographies may have biased the results to include more studies comparing groups of differing advantage, inflating the percentage of how many studies examined equigenesis within the larger context of the nature, children, and psychological health literature. Considering the numerous studies in the last few decades examining the nature-health relationship, the ratio of studies testing equigenesis to those not testing equigenesis within our set of selected studies is probably not representative of the ratio within the context of the larger body of literature. Our search only represents a small portion of the nature-health studies that have been published, which we feel only highlights the need for more studies exploring equigenic effects. Additionally, two of the searches included the key words “equigenesis” or “equigenic,” which may have biased the results to include more studies with positive equigenic effects compared to studies with no effect or effects in the opposite direction. The other two search phrases did not include “equigenesis” or “equigenic,” thus removing that bias of direction of effect. However, those searches included the key word “children,” which then excluded studies with adults. Thus, our sample of studies with adults is potentially more biased towards those with positive findings of equigenic effects. Furthermore, this review does not attempt to synthesize studies’ magnitudes of effect sizes or risks of bias. However, as one of the first studies to synthesize the relatively sparse literature testing for equigenic effects on children’s psychological health, it does provide a foundational collection of knowledge from which future empirical studies can design more rigorous evaluations. Future literature reviews could extend this review through analysis of methodological weaknesses and effect sizes in the body of literature.

### 4.5 Future research suggestions

As the concept of equigenesis has only recently been included in environmental psychology and health equity research, more research is required to better understand it. It is common for studies to “control for” measures of advantage when testing for the benefits of nature exposure in child development. However, more studies should include a direct comparison of outcomes between groups of differing advantage to reveal if nature exposure has the potential to support children with disadvantage more, lifting them to outcomes comparable to their advantaged peers. Advantage groups should be compared with respect to the effect size of a nature-based intervention or their outcomes in association with nature exposure. Of particular value would be longitudinal studies examining if inequalities narrow over time from nature exposure.

Future studies could strengthen measures of nature exposure, sampling of groups, and testing of mechanisms. For example, if nature exposure has an equigenic effect for children of disadvantage, under what conditions is it most effective? Direct measures of nature exposure (frequency of visits, total minutes of use, etc.) and analysis of how much nature exposure is enough to obtain positive outcomes for both groups (a dose–response relationship) may more accurately inform how nature exposure benefits psychological health for all children (Feng and Astell-Burt, 2017; Jimenez et al., 2022). There is also a need for study samples to compare equal-sized groups of advantaged and disadvantaged as well as employ more representative samples of all groups, which would increase generalizability and statistical power (McEachan et al., 2018). Replication studies involving the refinement and standardization of methods would allow for the integration of comparable results across studies, as heterogenous methods in the field have led to heterogenous results (Nigg et al., 2022). Alternatively, the use of many methods and tools through replication may establish robust findings for equigenesis (McEachan et al., 2018). Lastly, future research should continue to explore potential pathways, mechanisms, and moderators behind equigenesis, such as greenspace quality, parental satisfaction, different greenness types, access, and engagement (McCrorie et al., 2021; McEachan et al., 2018; Richardson et al., 2017).

### 4.6 Implications and applications

The implications of this work apply to various groups of adults that support children’s well-being. For example, parents can—and should—make regular time in their families’ schedules for time in nature. This can look like having “family time” in greenspaces, occasional trips to natural areas, and the reprioritization of time and family scheduling. If parents do not have the personal time to be outside with their children, they can find other chaperones or have their children go outside with a group of friends. If time outside of school is limited, parents may try to maximize nature exposure during the school day by seeking schools that emphasize nature-based learning and time in greenspaces or advocating for more nature exposure at school. Parents can also “bring nature inside” by adding plants and pets in their home.

However, as many of those interventions are less available to disadvantaged families, it is important for other adults to be involved as well in getting children the nature exposure they require. Policymakers and educators in disadvantaged areas can work toward implementing nature-based learning and green schoolyards, and communities and school boards can support funding of nature-based interventions (Sivarajah et al., 2018). Schools can also incorporate nature-based activities in their curricula, as well as time for walks and recess in greenspaces, including greenspaces beyond the schoolyard. Educators can also map out local greenspaces that the school could utilize for outdoor learning as well as consider having plants and small pets in their classrooms to promote attention restoration and stress reduction (Bernardo et al., 2021; Bølling et al., 2019).

The findings indicating that children living in disadvantaged circumstances benefit more from greenspace exposure have significant implications for policymaking regarding how and for whom resources are distributed or managed (e.g., schoolyard greenspaces) to support children’s health. Policymakers and urban planners should bring more natural areas closer to where children live, especially disadvantaged children, which Pérez-del-Pulgar et al. (2021) describe as “equitable place-based intervention.” Greenspaces close to where children live is important for reducing psychological inequalities as greenspaces closer to home are more likely to have bigger effects for disadvantaged children, who are more likely to be tied to their neighborhoods due to lack of mobility. Not only does location matter, but also the characteristics of the greenspaces, which should be of high quality and designed with intention. For example, these greenspaces should address the needs of all local communities (McEachan et al., 2018). They should also be able to encourage both active and passive activities—compatibility with users’ needs, a tenet of ART describing supportive environments (Kaplan, 1995)—so that children can experience psychological benefits through activities of their choosing (Caryl et al., 2024). And, as mentioned previously, specifically green schoolyards can reach wider audiences of children. Aside from physically implementing greenspaces, policymakers can also collaborate with public health officials to bring the importance of nature exposure to public awareness—especially for disadvantaged populations—and work to increase neighborhood safety so that residents feel more comfortable visiting their local greenspaces (Caryl et al., 2024; Putra et al., 2021).

Lastly, child psychologists, therapists, and pediatricians can incorporate nature into their practices as a way to improve mental health and psychological development. This may include participating in “nature prescription” programs such as ParkRx, working with community outreach programs centered around nature, or simply holding sessions outside in greenspaces (Rigolon et al., 2021).

## 5 Conclusion

This scoping review explored the state of research literature demonstrating equigenic effects on health associated with nature exposure, particularly children’s mental health and psychological development. We presented a broad look at equigenesis for all age groups and health outcomes; among those studies, there appears to be support for the equigenesis hypothesis. Of the relatively few studies examining children’s psychological health, support for equigenesis was detected for a variety of psychological outcomes, but mixed findings were also present, such as with studies that used multiple measures to assess a given variable and found variation in their results. Many questions remain, thus warranting further study of equigenesis. The findings to date imply that nature exposure could be a useful tool in promoting equity in psychological health among children of differing advantage. Communities and families should incorporate nature-based interventions in children’s daily lives to create equitable access and to reduce disparities in children’s mental health and psychological development.
