# Supplementary material for: Nature is nurture: a scoping review of nature exposure as an equigenic intervention on children’s psychological health
Source: Front Psychol. 2026 Apr 10;17:1731222. doi: 10.3389/fpsyg.2026.1731222 (PMC13105929; doi:10.3389/fpsyg.2026.1731222)
Supplement: Supplementary file 1 [file Table_1.docx]

Supplementary Material

# Supplementary Data

## Empirical Studies Meeting Inclusion Criteria, All Age Groups and Health Outcomes (*n* = 123)

1. Allard-Poesi, F., & Massu, J. (2023). Research Note: Is urban nature necessary for well-being? For whom? A necessary condition analysis. *Landscape and Urban Planning*, *234*, 104728. https://doi.org/10.1016/j.landurbplan.2023.104728
2. Amoly, E., Dadvand, P., Forns, J., López-Vicente, M., Basagaña, X., Julvez, J., Alvarez-Pedrerol, M., Nieuwenhuijsen, M. J., & Sunyer, J. (2014). Green and Blue Spaces and Behavioral Development in Barcelona Schoolchildren: The BREATHE Project. *Environmental Health Perspectives*, *122*(12), 1351–1358. https://doi.org/10.1289/ehp.1408215
3. Astell-Burt, T., & Feng, X. (2017). Does the Potential Benefit of Neighbourhood Green Space for Body Mass Index Depend upon Socioeconomic Circumstances and Local Built and Transport Environments? A Test of the ‘Equigenesis’ Hypothesis in Australia. *Journal of Transport & Health*, *5*, S40. https://doi.org/10.1016/j.jth.2017.05.327
4. Astell-Burt, T., Hartig, T., Eckermann, S., Nieuwenhuijsen, M., McMunn, A., Frumkin, H., & Feng, X. (2022). More green, less lonely? A longitudinal cohort study. *International Journal of Epidemiology*, *51*(1), 99–110. https://doi.org/10.1093/ije/dyab089
5. Balseviciene, B., Sinkariova, L., Grazuleviciene, R., Andrusaityte, S., Uzdanaviciute, I., Dedele, A., & Nieuwenhuijsen, M. (2014). Impact of Residential Greenness on Preschool Children’s Emotional and Behavioral Problems. *International Journal of Environmental Research and Public Health*, *11*(7), 6757–6770. https://doi.org/10.3390/ijerph110706757
6. Bao, W.-W., Jiang, N., Zhao, Y., Yang, B., Chen, G., Pu, Y., Ma, H., Liang, J., Xiao, X., Guo, Y., Dong, G., & Chen, Y. (2024). Urban greenspaces and child blood pressure in China: Evidence from a large population-based cohort study. *Environmental Research*, *244*, 117943. https://doi.org/10.1016/j.envres.2023.117943
7. Belcher, R. N., Murray, K. A., Reeves, J. P., & Fecht, D. (2024). Socioeconomic deprivation modifies green space and mental health associations: A within person study. *Environment International*, *192*, 109036. https://doi.org/10.1016/j.envint.2024.109036
8. Bernardo, F., Loupa-Ramos, I., Matos Silva, C., & Manso, M. (2021). The Restorative Effect of the Presence of Greenery on the Classroom in Children’s Cognitive Performance. *Sustainability*, *13*(6), 3488. https://doi.org/10.3390/su13063488
9. Boers, S., Hagoort, K., Scheepers, F., & Helbich, M. (2018). Does Residential Green and Blue Space Promote Recovery in Psychotic Disorders? A Cross-Sectional Study in the Province of Utrecht, The Netherlands. *International Journal of Environmental Research and Public Health*, *15*(10), 2195. https://doi.org/10.3390/ijerph15102195
10. Bolanis, D., Orri, M., Vergunst, F., Bouchard, S., Robitaille, É., Philippe, F., Ouellet-Morin, I., Girard, A., Paquin, V., Gauvin, L., Côté, S., & Geoffroy, M.-C. (2023). Increased urban greenspace in childhood associated with lower inattention deficit among adolescents. *Social Psychiatry and Psychiatric Epidemiology*, *59*(6), 947–956. https://doi.org/10.1007/s00127-023-02575-0
11. Bølling, M., Niclasen, J., Bentsen, P., & Nielsen, G. (2019). Association of Education Outside the Classroom and Pupils’ Psychosocial Well‐Being: Results From a School Year Implementation. *Journal of School Health*, *89*(3), 210–218. https://doi.org/10.1111/josh.12730
12. Browning, M. H. E. M., Li, D., White, M. P., Bratman, G. N., Becker, D., & Benfield, J. A. (2022). Association between residential greenness during childhood and trait emotional intelligence during young adulthood: A retrospective life course analysis in the United States. *Health & Place*, *74*, 102755. https://doi.org/10.1016/j.healthplace.2022.102755
13. Caryl, F., McCrorie, P., Olsen, J. R., & Mitchell, R. (2024). Use of natural environments is associated with reduced inequalities in child mental wellbeing: A cross-sectional analysis using global positioning system (GPS) data. *Environment International*, *190*, 108847. https://doi.org/10.1016/j.envint.2024.108847
14. Chang, H.-T., Wu, C.-D., Wang, J.-D., Chen, P.-S., & Su, H.-J. (2021). Residential green space structures are associated with a lower risk of bipolar disorder: A nationwide population-based study in Taiwan. *Environmental Pollution*, *283*, 115864. https://doi.org/10.1016/j.envpol.2020.115864
15. Cherrie, M. P. C., Shortt, N. K., Mitchell, R. J., Taylor, A. M., Redmond, P., Thompson, C. W., Starr, J. M., Deary, I. J., & Pearce, J. R. (2018). Green space and cognitive ageing: A retrospective life course analysis in the Lothian Birth Cohort 1936. *Social Science & Medicine*, *196*, 56–65. https://doi.org/10.1016/j.socscimed.2017.10.038
16. Christian, H., Ball, S. J., Zubrick, S. R., Brinkman, S., Turrell, G., Boruff, B., & Foster, S. (2017). Relationship between the neighbourhood built environment and early child development. *Health & Place*, *48*, 90–101. https://doi.org/10.1016/j.healthplace.2017.08.010
17. Cohen-Cline, H., Turkheimer, E., & Duncan, G. E. (2015). Access to green space, physical activity and mental health: a twin study. *Journal of Epidemiology and Community Health*, *69*(6), 523–529. https://doi.org/10.1136/jech-2014-204667
18. Cole, H. V. S., Triguero-Mas, M., Connolly, J. J. T., & Anguelovski, I. (2019). Determining the health benefits of green space: Does gentrification matter? *Health & Place*, *57*, 1–11. https://doi.org/10.1016/j.healthplace.2019.02.001
19. Dadvand, P., Hariri, S., Abbasi, B., Heshmat, R., Qorbani, M., Motlagh, M. E., Basagaña, X., & Kelishadi, R. (2019). Use of green spaces, self-satisfaction and social contacts in adolescents: A population-based CASPIAN-V study. *Environmental Research*, *168*, 171–177. https://doi.org/10.1016/j.envres.2018.09.033
20. Dadvand, P., Pujol, J., Macià, D., Martínez-Vilavella, G., Blanco-Hinojo, L., Mortamais, M., Alvarez-Pedrerol, M., Fenoll, R., Esnaola, M., Dalmau-Bueno, A., López-Vicente, M., Basagaña, X., Jerrett, M., Nieuwenhuijsen, M. J., & Sunyer, J. (2018). The Association between Lifelong Greenspace Exposure and 3-Dimensional Brain Magnetic Resonance Imaging in Barcelona Schoolchildren. *Environmental Health Perspectives*, *126*(2). https://doi.org/10.1289/ehp1876
21. Dadvand, P., Sunyer, J., Basagaña, X., Ballester, F., Lertxundi, A., Fernández-Somoano, A., Estarlich, M., García-Esteban, R., Mendez, M. A., & Nieuwenhuijsen, M. J. (2012). Surrounding Greenness and Pregnancy Outcomes in Four Spanish Birth Cohorts. *Environmental Health Perspectives*, *120*(10), 1481–1487. https://doi.org/10.1289/ehp.1205244
22. de la Osa, N., Navarro, J.-B., Penelo, E., Valentí, A., Ezpeleta, L., & Dadvand, P. (2024). Long-term exposure to greenspace and anxiety from preschool and primary school children. *Journal of Environmental Psychology*, *93*, 102207. https://doi.org/10.1016/j.jenvp.2023.102207
23. de Vries, S., Verheij, R. A., Groenewegen, P. P., & Spreeuwenberg, P. (2003). Natural Environments—Healthy Environments? An Exploratory Analysis of the Relationship between Greenspace and Health. *Environment and Planning A: Economy and Space*, *35*(10), 1717–1731. https://doi.org/10.1068/a35111
24. Dong, Y., Liu, H., & Zheng, T. (2021). Association between Green Space Structure and the Prevalence of Asthma: A Case Study of Toronto. *International Journal of Environmental Research and Public Health*, *18*(11), 5852. https://doi.org/10.3390/ijerph18115852
25. Dzhambov, A. M., Markevych, I., Hartig, T., Tilov, B., Arabadzhiev, Z., Stoyanov, D., Gatseva, P., & Dimitrova, D. D. (2018). Multiple pathways link urban green- and bluespace to mental health in young adults. *Environmental Research*, *166*, 223–233. https://doi.org/10.1016/j.envres.2018.06.004
26. Elliott, L. R., Pasanen, T., White, M. P., Wheeler, B. W., Grellier, J., Cirach, M., Bratman, G. N., van den Bosch, M., Roiko, A., Ojala, A., Nieuwenhuijsen, M., & Fleming, L. E. (2023). Nature contact and general health: Testing multiple serial mediation pathways with data from adults in 18 countries. *Environment International*, *178*, 108077. https://doi.org/10.1016/j.envint.2023.108077
27. Engemann, K., Pedersen, C. B., Agerbo, E., Arge, L., Børglum, A. D., Erikstrup, C., Hertel, O., Hougaard, D. M., McGrath, J. J., Mors, O., Mortensen, P. B., Nordentoft, M., Sabel, C. E., Sigsgaard, T., Tsirogiannis, C., Vilhjálmsson, B. J., Werge, T., Svenning, J.-C., & Horsdal, H. T. (2020). Association Between Childhood Green Space, Genetic Liability, and the Incidence of Schizophrenia. *Schizophrenia Bulletin*, *46*(6), 1629–1637. https://doi.org/10.1093/schbul/sbaa058
28. Engemann, K., Pedersen, C. B., Arge, L., Tsirogiannis, C., Mortensen, P. B., & Svenning, J.-C. (2018). Childhood exposure to green space – A novel risk-decreasing mechanism for schizophrenia? *Schizophrenia Research*, *199*, 142–148. https://doi.org/10.1016/j.schres.2018.03.026
29. Engemann, K., Pedersen, C. B., Arge, L., Tsirogiannis, C., Mortensen, P. B., & Svenning, J.-C. (2019). Residential green space in childhood is associated with lower risk of psychiatric disorders from adolescence into adulthood. *Proceedings of the National Academy of Sciences*, *116*(11), 5188–5193. https://doi.org/10.1073/pnas.1807504116
30. Engemann, K., Svenning, J.-C., Arge, L., Brandt, J., Bruun, M. T., Didriksen, M., Erikstrup, C., Geels, C., Hertel, O., Horsdal, H. T., Kaspersen, K. A., Mikkelsen, S., Mortensen, P. B., Nielsen, K. R., Ostrowski, S. R., Pedersen, O. B., Tsirogiannis, C., Sabel, C. E., Sigsgaard, T., … Pedersen, C. B. (2021). A life course approach to understanding associations between natural environments and mental well-being for the Danish blood donor cohort. *Health & Place*, *72*, 102678. https://doi.org/10.1016/j.healthplace.2021.102678
31. Engemann, K., Svenning, J.-C., Arge, L., Brandt, J., Erikstrup, C., Geels, C., Hertel, O., Mortensen, P. B., Plana-Ripoll, O., Tsirogiannis, C., Sabel, C. E., Sigsgaard, T., & Pedersen, C. B. (2020). Associations between growing up in natural environments and subsequent psychiatric disorders in Denmark. *Environmental Research*, *188*, 109788. https://doi.org/10.1016/j.envres.2020.109788
32. Ernst, J., Sobel, D., & Neil, A. (2022). Executive function in early childhood: Harnessing the potential of nature-based practices to elevate and equalize outcomes. *Frontiers in Education*, *7*. https://doi.org/10.3389/feduc.2022.1011912
33. Ernst, J., & Stelley, H. (2024). Supporting Young Children’s Self-Regulation Through Nature-Based Practices in Preschool. *Behavioral Sciences*, *14*(11), 1013. https://doi.org/10.3390/bs14111013
34. Ezpeleta, L., Navarro, J. B., Alonso, L., de la Osa, N., Ambrós, A., Ubalde, M., Penelo, E., & Dadvand, P. (2022). Greenspace Exposure and Obsessive-Compulsive Behaviors in Schoolchildren. *Environment and Behavior*, *54*(5), 893–916. https://doi.org/10.1177/00139165221095384
35. Feng, X., & Astell-Burt, T. (2017a). Do greener areas promote more equitable child health? *Health & Place*, *46*, 267–273. https://doi.org/10.1016/j.healthplace.2017.05.006
36. Feng, X., & Astell-Burt, T. (2017b). Residential Green Space Quantity and Quality and Child Well-being: A Longitudinal Study. *American Journal of Preventive Medicine*, *53*(5), 616–624. https://doi.org/10.1016/j.amepre.2017.06.035
37. Feng, X., & Astell-Burt, T. (2017c). The Relationship between Neighbourhood Green Space and Child Mental Wellbeing Depends upon Whom You Ask: Multilevel Evidence from 3083 Children Aged 12–13 Years. *International Journal of Environmental Research and Public Health*, *14*(3), 235. https://doi.org/10.3390/ijerph14030235
38. Feng, X., Astell-Burt, T., Standl, M., Flexeder, C., Heinrich, J., & Markevych, I. (2022). Green space quality and adolescent mental health: do personality traits matter? *Environmental Research*, *206*, 112591. https://doi.org/10.1016/j.envres.2021.112591
39. Fernandes, A., Avraam, D., Cadman, T., Dadvand, P., Guxens, M., Binter, A.-C., de Moira, A., Nieuwenhuijsen, M., Duijts, L., Julvez, J., de Castro, M., Fossati, S., Márquez, S., Vrijkotte, T., Elhakeem, A., McEachan, R., Yang, T., Pedersen, M., Vinther, J., … Vrijheid, M. (2024). Green spaces and respiratory, cardiometabolic, and neurodevelopmental outcomes: An individual-participant data meta-analysis of >35.000 European children. *Environment International*, *190*, 108853. https://doi.org/10.1016/j.envint.2024.108853
40. Fian, L., White, M. P., Arnberger, A., Thaler, T., Heske, A., & Pahl, S. (2024). Nature visits, but not residential greenness, are associated with reduced income-related inequalities in subjective well-being. *Health & Place*, *85*, 103175. https://doi.org/10.1016/j.healthplace.2024.103175
41. Flouri, E., Midouhas, E., & Joshi, H. (2014). The role of urban neighbourhood green space in children’s emotional and behavioural resilience. *Journal of Environmental Psychology*, *40*, 179–186. https://doi.org/10.1016/j.jenvp.2014.06.007
42. Garrett, J. K., Rowney, F. M., White, M. P., Lovell, R., Fry, R. J., Akbari, A., Geary, R., Lyons, R. A., Mizen, A., Nieuwenhuijsen, M., Parker, C., Song, J., Stratton, G., Thompson, D. A., Watkins, A., White, J., Williams, S. A., Rodgers, S. E., & Wheeler, B. W. (2023). Visiting nature is associated with lower socioeconomic inequalities in well-being in Wales. *Scientific Reports*, *13*(1). https://doi.org/10.1038/s41598-023-35427-7
43. Geary, R. S., Thompson, D., Mizen, A., Akbari, A., Garrett, J. K., Rowney, F. M., Watkins, A., Lyons, R. A., Stratton, G., Lovell, R., Nieuwenhuijsen, M., Parker, S. C., Song, J., Tsimpida, D., White, J., White, M. P., Williams, S., Wheeler, B. W., Fry, R., & Rodgers, S. E. (2023). Ambient greenness, access to local green spaces, and subsequent mental health: a 10-year longitudinal dynamic panel study of 2·3 million adults in Wales. *The Lancet Planetary Health*, *7*(10), e809–e818. https://doi.org/10.1016/s2542-5196(23)00212-7
44. Geiger, S. J., White, M. P., Davison, S. M. C., Zhang, L., McMeel, O., Kellett, P., & Fleming, L. E. (2023). Coastal proximity and visits are associated with better health but may not buffer health inequalities. *Communications Earth & Environment*, *4*(1). https://doi.org/10.1038/s43247-023-00818-1
45. Generaal, E., Hoogendijk, E. O., Stam, M., Henke, C. E., Rutters, F., Oosterman, M., Huisman, M., Kramer, S. E., Elders, P. J. M., Timmermans, E. J., Lakerveld, J., Koomen, E., ten Have, M., de Graaf, R., Snijder, M. B., Stronks, K., Willemsen, G., Boomsma, D. I., Smit, J. H., & Penninx, B. W. J. H. (2019). Neighbourhood characteristics and prevalence and severity of depression: pooled analysis of eight Dutch cohort studies. *British Journal of Psychiatry*, *215*(2), 468–475. https://doi.org/10.1192/bjp.2019.100
46. Gianaros, P. J., Miller, P. L., Manuck, S. B., Kuan, D. C.-H., Rosso, A. L., Votruba-Drzal, E. E., & Marsland, A. L. (2023). Beyond Neighborhood Disadvantage: Local Resources, Green Space, Pollution, and Crime as Residential Community Correlates of Cardiovascular Risk and Brain Morphology in Midlife Adults. *Psychosomatic Medicine*, *85*(5), 378–388. https://doi.org/10.1097/psy.0000000000001199
47. Guite, H. F., Clark, C., & Ackrill, G. (2006). The impact of the physical and urban environment on mental well-being. *Public Health*, *120*(12), 1117–1126. https://doi.org/10.1016/j.puhe.2006.10.005
48. Guo, H., Zhang, S., You, Y., Zheng, Q., Zhu, L., Hong, X.-C., & Ho, H. C. (2025). Assessing the impact of street-level greenery on older adults’ emotional well-being: A longitudinal study of equigenic potential and socioeconomic disparities. *Building and Environment*, *267*, 112309. https://doi.org/10.1016/j.buildenv.2024.112309
49. Hartley, J. M., Stevenson, K. T., Pereira, S. R., Peterson, M. N., Lawson, D. F., & Martinez, B. (2023). How a marine debris environmental education program plays to strengths of linguistically diverse learners. *Frontiers in Education*, *7*. https://doi.org/10.3389/feduc.2022.1058864
50. Hazlehurst, M. F., Hajat, A., Tandon, P. S., Szpiro, A. A., Kaufman, J. D., Tylavsky, F. A., Hare, M. E., Sathyanarayana, S., Loftus, C. T., LeWinn, K. Z., Bush, N. R., & Karr, C. J. (2024). Associations of residential green space with internalizing and externalizing behavior in early childhood. *Environmental Health*, *23*(1). https://doi.org/10.1186/s12940-024-01051-9
51. Huynh, Q., Craig, W., Janssen, I., & Pickett, W. (2013). Exposure to public natural space as a protective factor for emotional well-being among young people in Canada. *BMC Public Health*, *13*(1). https://doi.org/10.1186/1471-2458-13-407
52. Ji, J. S., Zhu, A., Bai, C., Wu, C.-D., Yan, L., Tang, S., Zeng, Y., & James, P. (2019). Residential greenness and mortality in oldest-old women and men in China: a longitudinal cohort study. *The Lancet Planetary Health*, *3*(1), e17–e25. https://doi.org/10.1016/s2542-5196(18)30264-x
53. Jimenez, M. P., Shoaff, J., Kioumourtzoglou, M.-A., Korrick, S., Rifas-Shiman, S. L., Hivert, M.-F., Oken, E., & James, P. (2021). Early-Life Exposure to Green Space and Mid-Childhood Cognition in the Project Viva Cohort, Massachusetts. *American Journal of Epidemiology*, *191*(1), 115–125. https://doi.org/10.1093/aje/kwab209
54. Kabisch, N., Haase, D., & van den Bosch, M. (2016). Adding Natural Areas to Social Indicators of Intra-Urban Health Inequalities among Children: A Case Study from Berlin, Germany. *International Journal of Environmental Research and Public Health*, *13*(8), 783. https://doi.org/10.3390/ijerph13080783
55. Kley, S., & Dovbischuk, T. (2024). The equigenic potential of green window views for city dwellers’ well-being. *Sustainable Cities and Society*, *108*, 105511. https://doi.org/10.1016/j.scs.2024.105511
56. Koh, C., Kondo, M. C., Rollins, H., & Bilal, U. (2022). Socioeconomic Disparities in Hypertension by Levels of Green Space Availability: A Cross-Sectional Study in Philadelphia, PA. *International Journal of Environmental Research and Public Health*, *19*(4), 2037. https://doi.org/10.3390/ijerph19042037
57. Kruize, H., van Kamp, I., van den Berg, M., van Kempen, E., Wendel-Vos, W., Ruijsbroek, A., Swart, W., Maas, J., Gidlow, C., Smith, G., Ellis, N., Hurst, G., Masterson, D., Triguero-Mas, M., Cirach, M., Gražulevičienė, R., van den Hazel, P., & Nieuwenhuijsen, M. (2020). Exploring mechanisms underlying the relationship between the natural outdoor environment and health and well-being – Results from the PHENOTYPE project. *Environment International*, *134*, 105173. https://doi.org/10.1016/j.envint.2019.105173
58. Kuo, M., Browning, M. H. E. M., Sachdeva, S., Lee, K., & Westphal, L. (2018). Might School Performance Grow on Trees? Examining the Link Between “Greenness” and Academic Achievement in Urban, High-Poverty Schools. *Frontiers in Psychology*, *9*. https://doi.org/10.3389/fpsyg.2018.01669
59. LeClair, J. A. (2024). Parental evaluations of neighbourhood green and play spaces and children’s mental health. *Canadian Geographies / Géographies Canadiennes*, *68*(3), 410–417. https://doi.org/10.1111/cag.12903
60. Maas, J., Verheij, R. A., de Vries, S., Spreeuwenberg, P., Schellevis, F. G., & Groenewegen, P. P. (2009). Morbidity is related to a green living environment. *Journal of Epidemiology & Community Health*, *63*(12), 967–973. https://doi.org/10.1136/jech.2008.079038
61. Markevych, I., Fuertes, E., Tiesler, C. M. T., Birk, M., Bauer, C.-P., Koletzko, S., von Berg, A., Berdel, D., & Heinrich, J. (2014). Surrounding greenness and birth weight: Results from the GINIplus and LISAplus birth cohorts in Munich. *Health & Place*, *26*, 39–46. https://doi.org/10.1016/j.healthplace.2013.12.001
62. Markevych, I., Tesch, F., Datzmann, T., Romanos, M., Schmitt, J., & Heinrich, J. (2018). Outdoor air pollution, greenspace, and incidence of ADHD: A semi-individual study. *Science of The Total Environment*, *642*, 1362–1368. https://doi.org/10.1016/j.scitotenv.2018.06.167
63. Markevych, I., Thiering, E., Fuertes, E., Sugiri, D., Berdel, D., Koletzko, S., von Berg, A., Bauer, C.-P., & Heinrich, J. (2014). A cross-sectional analysis of the effects of residential greenness on blood pressure in 10-year old children: results from the GINIplus and LISAplus studies. *BMC Public Health*, *14*(1), 477. https://doi.org/10.1186/1471-2458-14-477
64. Markevych, I., Tiesler, C. M. T., Fuertes, E., Romanos, M., Dadvand, P., Nieuwenhuijsen, M. J., Berdel, D., Koletzko, S., & Heinrich, J. (2014). Access to urban green spaces and behavioural problems in children: Results from the GINIplus and LISAplus studies. *Environment International*, *71*, 29–35. https://doi.org/10.1016/j.envint.2014.06.002
65. McCree, M., Cutting, R., & Sherwin, D. (2018). The Hare and the Tortoise go to Forest School: taking the scenic route to academic attainment via emotional wellbeing outdoors. *Early Child Development and Care*, *188*(7), 980–996. https://doi.org/10.1080/03004430.2018.1446430
66. McCrorie, P., Olsen, J. R., Caryl, F. M., Nicholls, N., & Mitchell, R. (2021). Neighbourhood natural space and the narrowing of socioeconomic inequality in children’s social, emotional, and behavioural wellbeing. *Wellbeing, Space and Society*, *2*, 100051. https://doi.org/10.1016/j.wss.2021.100051
67. McEachan, R. R. C., Yang, T. C., Roberts, H., Pickett, K. E., Arseneau-Powell, D., Gidlow, C. J., Wright, J., & Nieuwenhuijsen, M. (2018). Availability, use of, and satisfaction with green space, and children’s mental wellbeing at age 4 years in a multicultural, deprived, urban area: results from the Born in Bradford cohort study. *The Lancet Planetary Health*, *2*(6), e244–e254. https://doi.org/10.1016/s2542-5196(18)30119-0
68. Miller, P., Coley, R. L., Blatt, L., Spielvogel, B., & Votruba-Drzal, E. (2024). Using fixed-effects analyses to examine how neighborhood structural, process, and physical characteristics predict children’s cognitive skills in a national cohort of elementary school students. *Journal of Educational Psychology*, *116*(6), 936–952. https://doi.org/10.1037/edu0000860
69. Mitchell, R. (2013). Is physical activity in natural environments better for mental health than physical activity in other environments? *Social Science & Medicine*, *91*, 130–134. https://doi.org/10.1016/j.socscimed.2012.04.012
70. Mitchell, R. J., Richardson, E. A., Shortt, N. K., & Pearce, J. R. (2015). Neighborhood Environments and Socioeconomic Inequalities in Mental Well-Being. *American Journal of Preventive Medicine*, *49*(1), 80–84. https://doi.org/10.1016/j.amepre.2015.01.017
71. Mitchell, R., & Popham, F. (2008). Effect of exposure to natural environment on health inequalities: an observational population study. *The Lancet*, *372*(9650), 1655–1660. https://doi.org/10.1016/s0140-6736(08)61689-x
72. Moran, M. R., Bilal, U., Dronova, I., Ju, Y., Gouveia, N., Caiaffa, W. T., Friche, A. A. de L., Moore, K., Miranda, J. J., & Rodríguez, D. A. (2021). The equigenic effect of greenness on the association between education with life expectancy and mortality in 28 large Latin American cities. *Health & Place*, *72*, 102703. https://doi.org/10.1016/j.healthplace.2021.102703
73. Nawrath, M., Elsey, H., & Dallimer, M. (2022). Why cultural ecosystem services matter most: Exploring the pathways linking greenspaces and mental health in a low-income country. *Science of The Total Environment*, *806*, 150551. https://doi.org/10.1016/j.scitotenv.2021.150551
74. Nicholls, N., Caryl, F., Olsen, J. R., & Mitchell, R. (2022). Neighbourhood natural space and the narrowing of socioeconomic inequality in years of life lost: a cross-sectional ecological analysis of the Scottish Burden of Disease. *Journal of Epidemiology and Community Health*, *76*(12), 976–983. https://doi.org/10.1136/jech-2022-219111
75. Nigg, C., Niessner, C., Burchartz, A., Woll, A., & Schipperijn, J. (2022). The geospatial and conceptual configuration of the natural environment impacts the association with health outcomes and behavior in children and adolescents. *International Journal of Health Geographics*, *21*(1). https://doi.org/10.1186/s12942-022-00309-0
76. Olsen, J. R., Nicholls, N., & Mitchell, R. (2019). Are urban landscapes associated with reported life satisfaction and inequalities in life satisfaction at the city level? A cross-sectional study of 66 European cities. *Social Science & Medicine*, *226*, 263–274. https://doi.org/10.1016/j.socscimed.2019.03.009
77. Pérez-del-Pulgar, C., Anguelovski, I., Cole, H. V. S., de Bont, J., Connolly, J., Baró, F., Díaz, Y., Fontán-Vela, M., Duarte-Salles, T., & Triguero-Mas, M. (2021). The relationship between residential proximity to outdoor play spaces and children’s mental and behavioral health: The importance of neighborhood socio-economic characteristics. *Environmental Research*, *200*, 111326. https://doi.org/10.1016/j.envres.2021.111326
78. Plans-Beriso, E., Gullon, P., Fontan-Vela, M., Franco, M., Perez-Gomez, B., Pollan, M., Cura-Gonzalez, I., & Bilal, U. (2024). Modifying effect of urban parks on socioeconomic inequalities in diabetes prevalence: a cross-sectional population study of Madrid City, Spain. *Journal of Epidemiology and Community Health*, *78*(6), 360–366. https://doi.org/10.1136/jech-2023-221198
79. Poulain, T., Sobek, C., Ludwig, J., Igel, U., Grande, G., Ott, V., Kiess, W., Körner, A., & Vogel, M. (2020). Associations of Green Spaces and Streets in the Living Environment with Outdoor Activity, Media Use, Overweight/Obesity and Emotional Wellbeing in Children and Adolescents. *International Journal of Environmental Research and Public Health*, *17*(17), 6321. https://doi.org/10.3390/ijerph17176321
80. Putra, I. G. N. E., Astell-Burt, T., Cliff, D. P., Vella, S. A., & Feng, X. (2021). Association between caregiver perceived green space quality and the development of prosocial behaviour from childhood to adolescence: Latent class trajectory and multilevel longitudinal analyses of Australian children over 10 years. *Journal of Environmental Psychology*, *74*, 101579. https://doi.org/10.1016/j.jenvp.2021.101579
81. Reuben, A., Arseneault, L., Belsky, D. W., Caspi, A., Fisher, H. L., Houts, R. M., Moffitt, T. E., & Odgers, C. (2019). Residential neighborhood greenery and children’s cognitive development. *Social Science & Medicine*, *230*, 271–279. https://doi.org/10.1016/j.socscimed.2019.04.029
82. Richardson, E. A., Pearce, J., Shortt, N. K., & Mitchell, R. (2017). The role of public and private natural space in children’s social, emotional and behavioural development in Scotland: A longitudinal study. *Environmental Research*, *158*, 729–736. https://doi.org/10.1016/j.envres.2017.07.038
83. Rivadeneyra, P., Favaro, A., Meneguzzo, P., & Pirotti, F. (2024). Do greener childhoods mean fewer eating disorders? Using satellite imagery to uncover insights. *ISPRS Annals of the Photogrammetry, Remote Sensing and Spatial Information Sciences*, *X-3–2024*, 355–362. https://doi.org/10.5194/isprs-annals-x-3-2024-355-2024
84. Rostami, R., Lamit, H., Khoshnava, S. M., & Rostami, R. (2014). The Role of Historical Persian Gardens on the Health Status of Contemporary Urban Residents: Gardens and Health Status of Contemporary Urban Residents. *EcoHealth*, *11*(3), 308–321. https://doi.org/10.1007/s10393-014-0939-6
85. Sanders, T., Feng, X., Fahey, P. P., Lonsdale, C., & Astell-Burt, T. (2015). The influence of neighbourhood green space on children’s physical activity and screen time: findings from the longitudinal study of Australian children. *International Journal of Behavioral Nutrition and Physical Activity*, *12*(1). https://doi.org/10.1186/s12966-015-0288-z
86. Scaioli, G., Squillacioti, G., Bersia, M., Bellisario, V., Borraccino, A., Bono, R., Dalmasso, P., & Lemma, P. (2023). The wellbeing of adolescents and the role of greenness: A cross-sectional study among Italian students. *Frontiers in Public Health*, *10*. https://doi.org/10.3389/fpubh.2022.1050533
87. Schild, C., Reed, E., Hingston, T., Dennis, C., & Wilson, A. (2016). Neighborhood Characteristics: Influences on Pain and Physical Function in Youth at Risk for Chronic Pain. *Children*, *3*(4), 35. https://doi.org/10.3390/children3040035
88. Schinasi, L. H., Kondo, M. C., Edwards, J., Clougherty, J. E., de Roos, A. J., & Bilal, U. (2023). Does Urban Greenspace Reduce Mortality Inequalities Based on Poverty, Race, or Both in Philadelphia, PA? *Journal of Urban Health*, *100*(4), 686–695. https://doi.org/10.1007/s11524-023-00748-5
89. Sharifi, F., Nygaard, A., & Stone, W. M. (2021). Heterogeneity in the subjective well-being impact of access to urban green space. *Sustainable Cities and Society*, *74*, 103244. https://doi.org/10.1016/j.scs.2021.103244
90. Sivarajah, S., Smith, S. M., & Thomas, S. C. (2018). Tree cover and species composition effects on academic performance of primary school students. *PLOS ONE*, *13*(2), e0193254. https://doi.org/10.1371/journal.pone.0193254
91. Solmi, M., Thompson, T., Cortese, S., Estradé, A., Agorastos, A., Radua, J., Dragioti, E., Vancampfort, D., Thygesen, L. C., Aschauer, H., Schlögelhofer, M., Aschauer, E., Schneeberger, A., Huber, C. G., Hasler, G., Conus, P., Cuénod, K. Q. do, von Känel, R., Arrondo, G., … Correll, C. U. (2025). Collaborative outcomes study on health and functioning during infection times (COH-FIT): Insights on modifiable and non-modifiable risk and protective factors for wellbeing and mental health during the COVID-19 pandemic from multivariable and network analyses. *European Neuropsychopharmacology*, *90*, 1–15. https://doi.org/10.1016/j.euroneuro.2024.07.010
92. Stenfors, C. U. D., Rådmark, L., Stengård, J., Klein, Y., Osika, W., & Magnusson Hanson, L. L. (2024). More green, less depressed: Residential greenspace is associated with lower antidepressant redemptions in a nationwide population-based study. *Landscape and Urban Planning*, *249*, 105109. https://doi.org/10.1016/j.landurbplan.2024.105109
93. Subiza-Pérez, M., García-Baquero, G., Fernández-Somoano, A., Riaño, I., González, L., Delgado-Saborit, J. M., Guxens, M., Fossati, S., Vrijheid, M., Fernandes, A., Ibarluzea, J., & Lertxundi, N. (2023). Social inequalities, green and blue spaces and mental health in 6–12 years old children participating in the INMA cohort. *Health & Place*, *83*, 103104. https://doi.org/10.1016/j.healthplace.2023.103104
94. Sugiyama, T., Villanueva, K., Knuiman, M., Francis, J., Foster, S., Wood, L., & Giles-Corti, B. (2016). Can neighborhood green space mitigate health inequalities? A study of socio-economic status and mental health. *Health & Place*, *38*, 16–21. https://doi.org/10.1016/j.healthplace.2016.01.002
95. Thygesen, M., Engemann, K., Holst, G. J., Hansen, B., Geels, C., Brandt, J., Pedersen, C. B., & Dalsgaard, S. (2020). The Association between Residential Green Space in Childhood and Development of Attention Deficit Hyperactivity Disorder: A Population-Based Cohort Study. *Environmental Health Perspectives*, *128*(12). https://doi.org/10.1289/ehp6729
96. Towe-Goodman, N., McArthur, K. L., Willoughby, M., Swingler, M. M., Wychgram, C., Just, A. C., Kloog, I., Bennett, D. H., Berry, D., Hazlehurst, M. F., James, P., Jimenez, M. P., Lai, J.-S., Leve, L. D., Gatzke-Kopp, L., Schweitzer, J. B., Bekelman, T. A., Calub, C., Carnell, S., … Goodman, W. B. (2024). Green Space and Internalizing or Externalizing Symptoms Among Children. *JAMA Network Open*, *7*(4), e245742. https://doi.org/10.1001/jamanetworkopen.2024.5742
97. Triguero-Mas, M., Dadvand, P., Cirach, M., Martínez, D., Medina, A., Mompart, A., Basagaña, X., Gražulevičienė, R., & Nieuwenhuijsen, M. J. (2015). Natural outdoor environments and mental and physical health: Relationships and mechanisms. *Environment International*, *77*, 35–41. https://doi.org/10.1016/j.envint.2015.01.012
98. Triguero-Mas, M., Donaire-Gonzalez, D., Seto, E., Valentín, A., Martínez, D., Smith, G., Hurst, G., Carrasco-Turigas, G., Masterson, D., van den Berg, M., Ambròs, A., Martínez-Íñiguez, T., Dedele, A., Ellis, N., Grazulevicius, T., Voorsmit, M., Cirach, M., Cirac-Claveras, J., Swart, W., … Nieuwenhuijsen, M. J. (2017). Natural outdoor environments and mental health: Stress as a possible mechanism. *Environmental Research*, *159*, 629–638. https://doi.org/10.1016/j.envres.2017.08.048
99. Tsomokos, D. I., Papachristou, E., Rakesh, D., & Flouri, E. (2024). Family poverty, neighbourhood greenspace and perinatal outcomes. *Archives of Disease in Childhood*, *109*(12), 1017–1024. https://doi.org/10.1136/archdischild-2024-327349
100. van Aart, C. J. C., Michels, N., Sioen, I., de Decker, A., Bijnens, E. M., Janssen, B. G., de Henauw, S., & Nawrot, T. S. (2018). Residential landscape as a predictor of psychosocial stress in the life course from childhood to adolescence. *Environment International*, *120*, 456–463. https://doi.org/10.1016/j.envint.2018.08.028
101. van den Berg, M., van Poppel, M., van Kamp, I., Andrusaityte, S., Balseviciene, B., Cirach, M., Danileviciute, A., Ellis, N., Hurst, G., Masterson, D., Smith, G., Triguero-Mas, M., Uzdanaviciute, I., Wit, P. de, Mechelen, W. van, Gidlow, C., Grazuleviciene, R., Nieuwenhuijsen, M. J., Kruize, H., & Maas, J. (2016). Visiting green space is associated with mental health and vitality: A cross-sectional study in four european cities. *Health & Place*, *38*, 8–15. https://doi.org/10.1016/j.healthplace.2016.01.003
102. Vos, S., Bijnens, E. M., Renaers, E., Croons, H., van der Stukken, C., Martens, D. S., Plusquin, M., & Nawrot, T. S. (2022). Residential green space is associated with a buffering effect on stress responses during the COVID-19 pandemic in mothers of young children, a prospective study. *Environmental Research*, *208*, 112603. https://doi.org/10.1016/j.envres.2021.112603
103. Wang, P., Meng, Y.-Y., Lam, V., & Ponce, N. (2019). Green space and serious psychological distress among adults and teens: A population-based study in California. *Health & Place*, *56*, 184–190. https://doi.org/10.1016/j.healthplace.2019.02.002
104. Wang, R., Browning, M. H. E. M., Qin, X., He, J., Wu, W., Yao, Y., & Liu, Y. (2022). Visible green space predicts emotion: Evidence from social media and street view data. *Applied Geography*, *148*, 102803. https://doi.org/10.1016/j.apgeog.2022.102803
105. Wang, R., Dong, G., Cao, M., Zhou, Y., & Dong, G.-H. (2024). Exploring “Equigenesis” in the Associations Between Green Space and Kidney Health Among Middle-Aged and Older Adults Using Street View Data. *Innovation in Aging*, *8*(1). https://doi.org/10.1093/geroni/igad130
106. Wang, R., Feng, Z., & Pearce, J. (2022). Neighbourhood greenspace quantity, quality and socioeconomic inequalities in mental health. *Cities*, *129*, 103815. https://doi.org/10.1016/j.cities.2022.103815
107. Wang, R., Feng, Z., Pearce, J., Liu, Y., & Dong, G. (2021). Are greenspace quantity and quality associated with mental health through different mechanisms in Guangzhou, China: A comparison study using street view data. *Environmental Pollution*, *290*, 117976. https://doi.org/10.1016/j.envpol.2021.117976
108. Ward, J. S., Duncan, J. S., Jarden, A., & Stewart, T. (2016). The impact of children’s exposure to greenspace on physical activity, cognitive development, emotional wellbeing, and ability to appraise risk. *Health & Place*, *40*, 44–50. https://doi.org/10.1016/j.healthplace.2016.04.015
109. Ward Thompson, C., Roe, J., Aspinall, P., Mitchell, R., Clow, A., & Miller, D. (2012). More green space is linked to less stress in deprived communities: Evidence from salivary cortisol patterns. *Landscape and Urban Planning*, *105*(3), 221–229. https://doi.org/10.1016/j.landurbplan.2011.12.015
110. Wei, D., Lu, Y., Wu, X., Ho, H. C., Wu, W., Song, J., & Wang, Y. (2023). Greenspace exposure may increase life expectancy of elderly adults, especially for those with low socioeconomic status. *Health & Place*, *84*, 103142. https://doi.org/10.1016/j.healthplace.2023.103142
111. Wei, D., Lu, Y., Zhou, Y., Ho, H. C., & Jiang, B. (2024). The effect of peri-urban parks on life expectancy and socioeconomic inequalities: A 16-year longitudinal study in Hong Kong. *Landscape and Urban Planning*, *252*, 105192. https://doi.org/10.1016/j.landurbplan.2024.105192
112. Wells, N. M., Myers, B. M., Todd, L. E., Barale, K., Gaolach, B., Ferenz, G., Aitken, M., Henderson, C. R., Tse, C., Pattison, K. O., Taylor, C., Connerly, L., Carson, J. B., Gensemer, A. Z., Franz, N. K., & Falk, E. (2015). The Effects of School Gardens on Children’s Science Knowledge: A randomized controlled trial of low-income elementary schools. *International Journal of Science Education*, *37*(17), 2858–2878. https://doi.org/10.1080/09500693.2015.1112048
113. Wheeler, B. W., Lovell, R., Higgins, S. L., White, M. P., Alcock, I., Osborne, N. J., Husk, K., Sabel, C. E., & Depledge, M. H. (2015). Beyond greenspace: an ecological study of population general health and indicators of natural environment type and quality. *International Journal of Health Geographics*, *14*(1). https://doi.org/10.1186/s12942-015-0009-5
114. White, M. P., Elliott, L. R., Grellier, J., Economou, T., Bell, S., Bratman, G. N., Cirach, M., Gascon, M., Lima, M. L., Lõhmus, M., Nieuwenhuijsen, M., Ojala, A., Roiko, A., Schultz, P. W., van den Bosch, M., & Fleming, L. E. (2021). Associations between green/blue spaces and mental health across 18 countries. *Scientific Reports*, *11*(1). https://doi.org/10.1038/s41598-021-87675-0
115. White, M. P., Pahl, S., Wheeler, B. W., Depledge, M. H., & Fleming, L. E. (2017). Natural environments and subjective wellbeing: Different types of exposure are associated with different aspects of wellbeing. *Health & Place*, *45*, 77–84. https://doi.org/10.1016/j.healthplace.2017.03.008
116. Wu, J., & Jackson, L. (2017). Inverse relationship between urban green space and childhood autism in California elementary school districts. *Environment International*, *107*, 140–146. https://doi.org/10.1016/j.envint.2017.07.010
117. Wu, W., Yun, Y., Zhai, J., Sun, Y., Zhang, G., & Wang, R. (2021). Residential self-selection in the greenness-wellbeing connection: A family composition perspective. *Urban Forestry & Urban Greening*, *59*, 127000. https://doi.org/10.1016/j.ufug.2021.127000
118. Yang, T., Barnett, R., Fan, Y., & Li, L. (2019). The effect of urban green space on uncertainty stress and life stress: A nationwide study of university students in China. *Health & Place*, *59*, 102199. https://doi.org/10.1016/j.healthplace.2019.102199
119. Younan, D., Tuvblad, C., Li, L., Wu, J., Lurmann, F., Franklin, M., Berhane, K., McConnell, R., Wu, A. H., Baker, L. A., & Chen, J.-C. (2016). Environmental Determinants of Aggression in Adolescents: Role of Urban Neighborhood Greenspace. *Journal of the American Academy of Child & Adolescent Psychiatry*, *55*(7), 591–601. https://doi.org/10.1016/j.jaac.2016.05.002
120. Yue, Y., Yang, D., & van Dyck, D. (2022). Urban greenspace and mental health in Chinese older adults: Associations across different greenspace measures and mediating effects of environmental perceptions. *Health & Place*, *76*, 102856. https://doi.org/10.1016/j.healthplace.2022.102856
121. Zach, A., Meyer, N., Hendrowarsito, L., Kolb, S., Bolte, G., Nennstiel-Ratzel, U., Stilianakis, N. I., & Herr, C. (2016). Association of sociodemographic and environmental factors with the mental health status among preschool children—Results from a cross-sectional study in Bavaria, Germany. *International Journal of Hygiene and Environmental Health*, *219*(4–5), 458–467. https://doi.org/10.1016/j.ijheh.2016.04.012
122. Zhao, Y., van den Berg, P. E. W., Ossokina, I. v, & Arentze, T. A. (2024). How do urban parks, neighborhood open spaces, and private gardens relate to individuals’ subjective well-being: Results of a structural equation model. *Sustainable Cities and Society*, *101*, 105094. https://doi.org/10.1016/j.scs.2023.105094
123. Zhong, C., Yin, X., Fallah-Shorshani, M., Islam, T., McConnell, R., Fruin, S., & Franklin, M. (2023). Disparities in greenspace associated with sleep duration among adolescent children in Southern California. *Environmental Epidemiology*, *7*(4), e264. https://doi.org/10.1097/ee9.0000000000000264

## Literature Review Articles Meeting Inclusion Criteria (*n* = 20)

1. Aghabozorgi, K., van der Jagt, A., Bell, S., & Brown, C. (2023). Assessing the impact of blue and green spaces on mental health of disabled children: A scoping review. *Health & Place*, *84*, 103141. https://doi.org/10.1016/J.HEALTHPLACE.2023.103141
2. Alderton, A., Villanueva, K., O’Connor, M., Boulangé, C., & Badland, H. (2019). Reducing Inequities in Early Childhood Mental Health: How Might the Neighborhood Built Environment Help Close the Gap? A Systematic Search and Critical Review. *International Journal of Environmental Research and Public Health*, *16*(9), 1516. https://doi.org/10.3390/ijerph16091516
3. Badland, H., & Pearce, J. (2019). Liveable for whom? Prospects of urban liveability to address health inequities. *Social Science & Medicine*, *232*, 94–105. https://doi.org/10.1016/J.SOCSCIMED.2019.05.001
4. Banay, R. F., Bezold, C. P., James, P., Hart, J. E., & Laden, F. (2017). Residential greenness: Current perspectives on its impact on maternal health and pregnancy outcomes. *International Journal of Women’s Health*, *9*, 133–144. https://doi.org/10.2147/IJWH.S125358
5. Bikomeye, J., Balza, J., & Beyer, K. (2021). The Impact of Schoolyard Greening on Children’s Physical Activity and Socioemotional Health: A Systematic Review of Experimental Studies. *International Journal of Environmental Research and Public Health*, *18*(2), 535. https://doi.org/10.3390/ijerph18020535
6. Christian, H., Zubrick, S. R., Foster, S., Giles-Corti, B., Bull, F., Wood, L., Knuiman, M., Brinkman, S., Houghton, S., & Boruff, B. (2015). The influence of the neighborhood physical environment on early child health and development: A review and call for research. *Health & Place*, *33*, 25–36. https://doi.org/10.1016/J.HEALTHPLACE.2015.01.005
7. Fyfe-Johnson, A. L., Hazlehurst, M. F., Perrins, S. P., Bratman, G. N., Thomas, R., Garrett, K. A., Hafferty, K. R., Cullaz, T. M., Marcuse, E. K., & Tandon, P. S. (2021). Nature and Children’s Health: A Systematic Review. *Pediatrics*, *148*(4). https://doi.org/10.1542/peds.2020-049155
8. Kuo, M., Barnes, M., & Jordan, C. (2019). Do Experiences With Nature Promote Learning? Converging Evidence of a Cause-and-Effect Relationship. *Frontiers in Psychology*, *10*. https://doi.org/10.3389/fpsyg.2019.00305
9. Leese, C., & Al‐Zubaidi, H. (2024). Urban green and blue spaces for influencing physical activity in the United Kingdom: A narrative review of the policy and evidence. *Lifestyle Medicine*, *5*(1). https://doi.org/10.1002/lim2.96
10. Luque-García, L., Corrales, A., Lertxundi, A., Díaz, S., & Ibarluzea, J. (2022). Does exposure to greenness improve children’s neuropsychological development and mental health? A Navigation Guide systematic review of observational evidence for associations. *Environmental Research*, *206*, 112599. https://doi.org/10.1016/J.ENVRES.2021.112599
11. Markevych, I., Schoierer, J., Hartig, T., Chudnovsky, A., Hystad, P., Dzhambov, A. M., de Vries, S., Triguero-Mas, M., Brauer, M., Nieuwenhuijsen, M. J., Lupp, G., Richardson, E. A., Astell-Burt, T., Dimitrova, D., Feng, X., Sadeh, M., Standl, M., Heinrich, J., & Fuertes, E. (2017). Exploring pathways linking greenspace to health: Theoretical and methodological guidance. *Environmental Research*, *158*, 301–317. https://doi.org/10.1016/J.ENVRES.2017.06.028
12. Moll, A., Collado, S., Staats, H., & Corraliza, J. A. (2022). Restorative effects of exposure to nature on children and adolescents: A systematic review. *Journal of Environmental Psychology*, *84*, 101884. https://doi.org/10.1016/J.JENVP.2022.101884
13. Rigolon, A., Browning, M. H. E. M., McAnirlin, O., & Yoon, H. (Violet). (2021). Green Space and Health Equity: A Systematic Review on the Potential of Green Space to Reduce Health Disparities. *International Journal of Environmental Research and Public Health*, *18*(5), 2563. https://doi.org/10.3390/ijerph18052563
14. Romanello, M., McGushin, A., di Napoli, C., Drummond, P., Hughes, N., Jamart, L., Kennard, H., Lampard, P., Solano Rodriguez, B., Arnell, N., Ayeb-Karlsson, S., Belesova, K., Cai, W., Campbell-Lendrum, D., Capstick, S., Chambers, J., Chu, L., Ciampi, L., Dalin, C., … Hamilton, I. (2021). The 2021 report of the Lancet Countdown on health and climate change: code red for a healthy future. *The Lancet*, *398*(10311), 1619–1662. https://doi.org/10.1016/S0140-6736(21)01787-6
15. Seastedt, H., Schuetz, J., Perkins, A., Gamble, M., & Sinkkonen, A. (2024). Impact of urban biodiversity and climate change on children’s health and well being. *Pediatric Research*. https://doi.org/10.1038/s41390-024-03769-1
16. Tillmann, S., Tobin, D., Avison, W., & Gilliland, J. (2018). Mental health benefits of interactions with nature in children and teenagers: a systematic review. *Journal of Epidemiology and Community Health*, *72*(10), 958–966. https://doi.org/10.1136/jech-2018-210436
17. Timar, E., Gromada, A., Rees, G., & Carraro, A. (2022). Places and spaces: Environments and children’s well-being. In *Innocenti Report Card 17*. UNICEF Office of Research – Innocenti.
18. Vanaken, G. J., & Danckaerts, M. (2018). Impact of green space exposure on children’s and adolescents’ mental health: A systematic review. *International Journal of Environmental Research and Public Health*, *15*(12). https://doi.org/10.3390/ijerph15122668
19. Zare Sakhvidi, M. J., Knobel, P., Bauwelinck, M., de Keijzer, C., Boll, L. M., Spano, G., Ubalde-Lopez, M., Sanesi, G., Mehrparvar, A. H., Jacquemin, B., & Dadvand, P. (2022). Greenspace exposure and children behavior: A systematic review. *Science of The Total Environment*, *824*, 153608. https://doi.org/10.1016/J.SCITOTENV.2022.153608
20. Zare Sakhvidi, M. J., Mehrparvar, A. H., Zare Sakhvidi, F., & Dadvand, P. (2023). Greenspace and health, wellbeing, physical activity, and development in children and adolescents: An overview of the systematic reviews. *Current Opinion in Environmental Science & Health*, *32*, 100445. https://doi.org/10.1016/J.COESH.2023.100445

# Supplementary Tables

**Supplementary Table 1.** Operational definitions of independent and dependent variables.

| ***Nature and greenspace:***  “Nature” encompasses any environment containing plants (trees, shrubs, grass, flowers, etc.), water, animals and insects, and other non-built elements. The term nature is commonly used synonymously with the term ‘greenspace’ or ‘green space.’ Greenspaces are typically defined as areas dominated by the presence of plants such as trees, shrubs, and grass, and contain few, if any, built structures, such as parks, forest preserves, etc. Empirical studies that analyze nature visits, time spent in nature, or distance to greenspace typically focus on those types of greenspaces (e.g. Caryl et al., 2024, Fian et al., 2024, Pérez-del-Pulgar et al., 2021).  Research also commonly defines nature exposure by quantifying “greenness”—the general presence of plant foliage (trees, shrubs, grass, etc.)—within a defined area, such as a neighborhood, schoolyard, or surrounding a residential address. The greenness or naturalness of the area is quantified through the use of satellite-based data such as greenness indices (e.g. NDVI) and land cover and land use data (LULC).  Other measures of nature exposure include “green window views” (e.g. Kley & Dovbischuk, 2024), indoor greenery (e.g. Bernardo et al., 2021), environmental educational programs (e.g. Bølling et al., 2019, Ernst & Stelley, 2024, Hartley et al., 2023), and perceived quality of accessible green spaces (e.g. Putra et al., 2021).  As this review is interested in the overall relationship between nature exposure and health outcomes for different groups of advantage, we included all definitions of nature and nature exposure in our study selection. However, we opted to use the term “green space” in our database search, as the term “nature” is often conflated with the broader definition (“inherent character or basic constitution of a person or thing,” Merriam-Webster), particularly in social science research. |
| --- |
| ***Psychological development:***  Psychological development encompasses the changes that occur over time in various psychological domains, such as the social, emotional, and cognitive (Berk, 2013). Psychological development is highly intertwined with mental health, as the processes among the various psychological domains affect and are affected by mental health status (e.g. someone who struggles socially is more likely to be depressed, etc.).  Berk, L. E. (2013). *Child development* (9th ed.). Pearson. |
| ***Mental health:***  Our definition of mental health aligns with the definition stated by the World Health Organization, which is that mental health is the “state of mental well-being that enables people to cope with the stresses of life, to realize their abilities, to learn well and work well, and to contribute to their communities” (World Health Organization, 2022). Our understanding of mental health encompasses overall mental patterns, such as general demeanor and how an individual is feeling about themselves and their life, either positively or negatively. As stated by the Substance Abuse and Mental Health Services Administration, mental health “affects how we think, feel, and act” (SAMHSA, 2023).  World Health Organization. (2022). *World mental health report: Transforming mental health for all*. World Health Organization. https://www.who.int/publications/i/item/9789240049338  Substance Abuse and Mental Health Services Administration. (2023, April 24). *What is mental health?* U.S. Department of Health & Human Services. https://www.samhsa.gov/mental-health/what-is-mental-health |

**Supplementary Table 2.** Criteria for the categorization of studies by health outcome.

| **Health Outcome Category** | **Criteria** |
| --- | --- |
| Positive Mental Health Outcomes | Studies assessing overall mental well-being, life satisfaction, sense of purpose, and self-satisfaction. |
| Negative Mental Health Outcomes | Studies assessing psychopathology or negative mental health symptoms, including depression, anxiety, stress, OCD, schizophrenia, symptoms of ADHD, etc. Studies may examine a multitude of psychopathological conditions or specific symptoms, such as feelings of loneliness. |
| Cognitive Functioning and Development | Studies assessing academic performance (reading and math skills), memory, attention, visual and verbal intelligence, or executive functioning. |
| Socioemotional Functioning and Development | Studies assessing socioemotional health and behaviors. This includes studies using the Strengths & Difficulties Questionnaire (SDQ) as well as studies assessing emotional regulation (self-regulation) or emotional intelligence. Note that the SDQ features a scale for prosocial behavior, but studies that used the entire SDQ were categorized under this category. |
| Prosocial Behavior | Studies assessing prosocial behavior as an individual measure. This includes using only the prosocial subscale from the SDQ or other measures assessing social behavior. |
| Pro-environmental Behavior and Attitudes | Studies assessing pro-environmental behaviors and attitudes. |
| Physical Health | Studies assessing any aspect of physical health, including but not limited to cardiovascular functioning, respiratory functioning, physical disease, weight, etc. |
| Physical Activity | Studies assessing the individual level of physical activity. Studies were only counted in this category when physical activity was the dependent variable, not the mediating or independent variable. |
| Pregnancy Outcomes | Studies assessing pregnancy outcomes. |
| Mortality and Life Expectancy | Studies assessing mortality and/or life expectancy. |
| Other | Studies assessing any other outcome not mentioned above (ex. sleep). |

# Supplementary Figures

**Supplementary Figure 1.** Study characteristics of the equigenic literature on children’s psychological health. **(A)** Studies organized by publication year. **(B)** Studies organized by study location. **(C)** Studies organized by psychological health outcome. Note: Figure 1c includes double counts, as some studies used multiple measures in their methods to assess various health outcomes.
